# Pandemic monitoring with global aircraft-based wastewater surveillance networks

**DOI:** 10.1038/s41591-025-03501-4

**Published:** 2025-02-12

**Authors:** Guillaume St-Onge, Jessica T. Davis, Laurent Hébert-Dufresne, Antoine Allard, Alessandra Urbinati, Samuel V. Scarpino, Matteo Chinazzi, Alessandro Vespignani

**Affiliations:** 1 Laboratory for the Modeling of Biological and Socio-technical Systems, Northeastern University, Boston, MA USA; 2 The Roux Institute, Northeastern University, Portland, ME USA; 3 Network Science Institute, Northeastern University, Boston, MA USA; 4 Vermont Complex Systems Institute, University of Vermont, Burlington, VT USA; 5 Department of Computer Science, University of Vermont, Burlington, VT USA; 6 Département de physique, de génie physique et d’optique, Université Laval, Québec City, Quebec Canada; 7 Centre interdisciplinaire en modélisation mathématique, Université Laval, Québec City, Quebec Canada; 8 Institute for Experiential AI, Northeastern University, Boston, MA USA; 9 Santa Fe Institute, Santa Fe, NM USA; 10 Institute for Scientific Interchange Foundation, Turin, Italy

**Keywords:** Epidemiology, Population screening, Infectious diseases, Computational models, Ecological epidemiology

## Abstract

Aircraft wastewater surveillance has been proposed as a new approach to monitor the global spread of pathogens. Here we develop a computational framework providing actionable information for the design and estimation of the effectiveness of global aircraft-based wastewater surveillance networks (WWSNs). We study respiratory diseases of varying transmission potential and find that networks of 10–20 strategically placed wastewater sentinel sites can provide timely situational awareness and function effectively as an early warning system. The model identifies potential blind spots and suggests optimization strategies to increase WWSN effectiveness while minimizing resource use. Our findings indicate that increasing the number of sentinel sites beyond a critical threshold does not proportionately improve WWSN capabilities, emphasizing the importance of resource optimization. We show, through retrospective analyses, that WWSNs can notably shorten detection time for emerging pathogens. The approach presented offers a realistic analytic framework for the analysis of WWSNs at airports.

## Abstract (web summary)

By simulating the implementation of airport-based wastewater surveillance sites at the global level, a modeling study shows how this early warning system would perform in identifying sources of pandemic outbreaks, in time and space, and what the optimal location of monitoring sites would be.

## Main

Recent health crises have called attention to the dual role of airports, both in the global spread of infectious diseases and acting as convenient frontlines for detection and monitoring of emerging health threats^1–5^. In this context, aircraft-based wastewater surveillance is gaining increasing scientific and operational interest as a noninvasive method to track pathogens. Traditionally, wastewater surveillance has been used to monitor community prevalence of pathogens such as SARS-CoV-2 variants, poliomyelitis and influenza^6–9^. Expanding wastewater surveillance at airports to create a global wastewater surveillance network (WWSN) has recently been proposed as a new early warning system against emerging pathogens^10–13^. Several national and international initiatives aim to operationalize aircraft wastewater surveillance to enhance global monitoring and early detection of emerging pathogens^14,15^. However, establishing a global WWSN presents challenges, including efficient sample collection, genomic analysis logistics, pathogen selection, optimal airport surveillance, network scaling and addressing blind spots to balance effectiveness and cost^16^. While there have been studies on the feasibility of aircraft wastewater surveillance at several major airports^17–20^, fully understanding the performance of a WWSN—in terms of its size, distributed locations and operations—remains to be addressed.

Here, we use the Global Epidemic and Mobility Model (GLEAM)^21–23^ to study the performance of global aircraft WWSNs, offering insights into pathogen spread and detection. GLEAM is a stochastic, spatial, age-structured metapopulation model dividing the global population into over 3,200 subpopulations across more than 200 countries and territories interconnected by air travel and commuting networks. Its air travel component, based on data from over 4,600 airports provided by the Official Aviation Guide (OAG) database, incorporates flight segments and origin–destination information (Methods and Supplementary Information 1). Coupled with an epidemic compartmental model tracking individuals within various disease stages (for example, susceptible, latent and infectious), GLEAM simulates the dissemination of a contagion across subpopulations. It has been used to model global health threats including pandemic influenza, Ebola, Zika and SARS-CoV-2 (refs. ^24–26^). To simulate a surveillance system within GLEAM, we create a global WWSN that consists of multiple surveillance sites termed sentinels. We assume that each sentinel airport will test the wastewater from a given fraction of international flight arrivals per day.

The model generates stochastic simulations of global epidemic spread from any initial outbreak conditions, producing daily data on infection importations (international and domestic), infection incidence and individual-level detections at sentinel sites. The early growth phase of the modeled epidemics can also be mapped onto a multitype branching process, enabling efficient computation of key analytics via probability-generating functions (PGFs). These analytics include the time to first detection and also, based on sentinel detections, source identification, reproduction number ($\mathcal{R}_0$) estimation and outbreak onset timing. Together, these metrics offer a framework for evaluation of the WWSN’s effectiveness in real time for surveillance and public health response.

## Results

There are multiple strategies for testing wastewater collected from aircraft for the presence of pathogens. Monitoring efforts can either target a specific pathogen or a priority list of pathogens, such as those identified by the World Health Organization R&D Blueprint, or search for new pathogens using untargeted metagenomic and metatranscriptomic sequencing^11,27^. Each of these strategies can be incorporated into our framework following model adjustment. In this study, we focus on the example of detecting SARS-CoV-2 variants using reverse-transcription quantitative polymerase chain reaction, with positive samples undergoing whole-genome sequencing to identify specific variants.

**Fig. 1:**
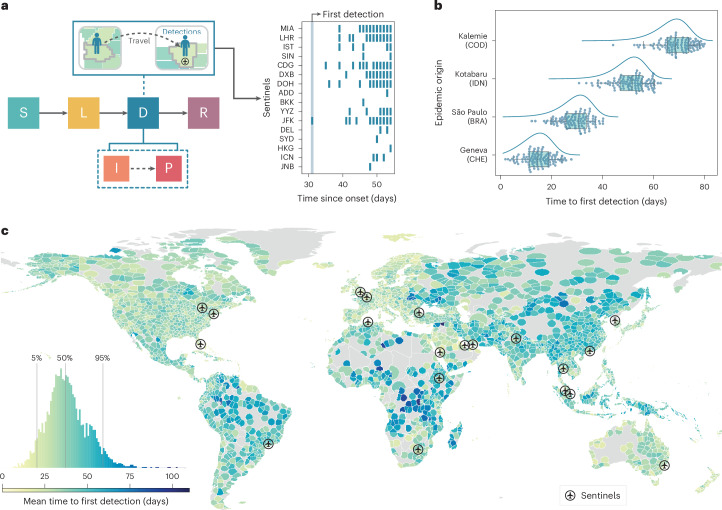
Time required to detect a new pathogen with a global surveillance network at airports. **a**–**c**, The surveillance network consists of 20 sentinel airports selected for high international passenger volume and geographical diversity (Supplementary Table 6). We use an average $\mathcal{R}_0$ = 2, at the source, *T*_gen_ of 4 days and a postinfectious period of 10 days, resulting in a detectable period of ~12.7 days. Detectable individuals have a 16% probability of detection on international flights to sentinels. **a**, Schematic of the SLDR model and an example of binary detection time series at sentinel airports, using São Paulo as the origin. Only sentinels (identified by IATA codes) with detections are shown. **b**, Time to first detection by the sentinel network for four origins (ISO 3166-1 alpha-3 country codes of the origins in parentheses). Dots represent GLEAM simulations (*n* = 100 for each origin), with boxplots summarizing the results: median (center line), interquartile range (box) and 90% central prediction interval (whiskers, 5th–95th percentle). Curves show analytical distributions from PGF methodology. **c**, *T*_fd_ by the network for outbreaks originating from each subpopulation. The histogram (lower left) compiles results from 3,244 subpopulations. Source data

We start our analysis by considering a baseline aircraft WWSN with 20 sentinel sites. To achieve sufficient regional coverage, we selected the three busiest international airports from each of the six World Health Organization regions and added two additional sites in South America and Oceania. The locations are shown, by airport markers, in Fig. 1 and are reported in Supplementary Table 6. We show in Supplementary Fig. 14 that selection of less busy airports generally delays disease detection, because larger international hubs offer broader coverage and more frequent flight connections, enabling faster detection.

The efficiency of the WWSN depends on the pathogen’s intrinsic characteristics, its detectability and the fraction of aircraft tested at sentinel sites. Here we consider a SARS-CoV-2-like respiratory infection with a wastewater detectability period consistent with reported values in the literature^6,28,29^. We map an individual’s disease history to a susceptible–latent–detectable–recovered (SLDR) compartmental structure, as shown in Fig. 1a. Susceptible (S) individuals can become infected through exposure to infectious individuals. Latent (L) individuals have been exposed but are not yet transmitting the pathogen and remain undetectable in the wastewater. Detectable (D) individuals include both infectious (I) individuals who can transmit the pathogen and postinfectious (P) individuals who no longer infect others but are still detectable through wastewater. Finally, recovered (R) individuals are no longer detectable and cannot be reinfected (Methods and Supplementary Information 1).

Each traveling detectable individual arriving at a sentinel on an international flight is detected with probability *p*_det_. The detection rate, *p*_det_, combines the fraction of sampled aircraft, the probability an individual uses the lavatory during a flight and the probability that a detectable individual is shedding sufficient virus to lead to a detection. Because current detectability estimates for SARS-CoV-2 in aircraft wastewater vary considerably^10,12,19^, our analysis varies *p*_det_ from 4 to 32%. This variation in probability accounts for different estimates of detectability in the wastewater and different fractions of flights tested (Methods includes a detailed discussion). While sampling of individual aircraft independently maximizes detection accuracy, testing combined wastewater at a consolidation point, such as an airport triturator, may be more cost effective. Thus we assume pooled sampling, in which multiple detectable individuals traveling through the same sentinel on the same day result in a single detection, producing binary detection time series as shown in Fig. 1a. It is worth noting that most of these assumptions can be adjusted to accommodate alternative detection schemes, sampling cadences and sentinel site locations.

### Baseline WWSN performance

A key metric for evaluating the effectiveness of a WWSN is the time to first detection of an emerging pathogen. This metric measures the number of days from an outbreak onset to the first detection at any sentinel. We simulate an epidemic seeded in one subpopulation with ten latent and ten infectious individuals. The time to first detection depends on the WWSN configuration, outbreak origin, pathogen traits, *p*_det_ and stochastic variations in travel and detection events.

**Fig. 2:**
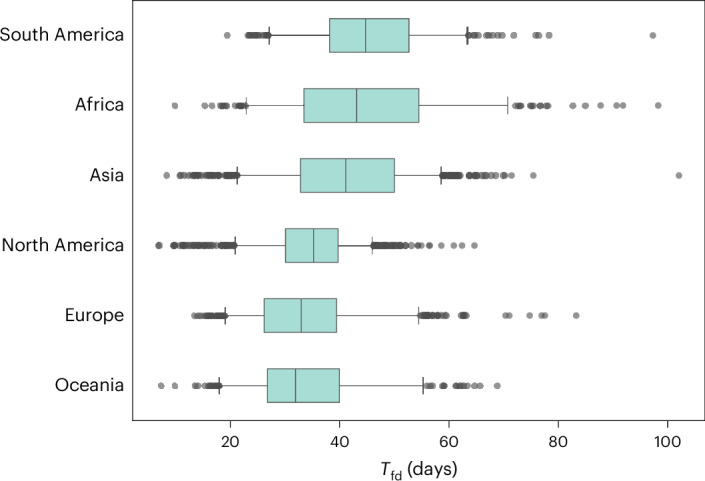
Heterogeneity of *T*_fd_ within geographical regions. We aggregate *T*_fd_ obtained from Fig. 1 over continents (South America, *n* = 297; Africa, *n* = 338; Asia, *n* = 867; North America, *n* = 854; Europe, *n* = 596; Oceania, *n* = 292). Boxplots show the median (center line), interquartile range (box), 90% central PI (whiskers, 5th–95th percentile) and outliers outside the interval (black dots). Numerical values for some statistics of *T*_fd_ are reported in Extended Data Table 1. Source data

In Fig. 1b, we show the full probability distribution for the time to first detection for four different origins: Geneva (Switzerland), São Paulo (Brazil), Kotabaru (Indonesia) and Kalemie (Democratic Republic of the Congo). The time to first detection varies widely, from a mean of 14.2 days (90% PI, 4–22) for Geneva to 66.5 days (90% PI, 53–76) for Kalemie, where PI is central prediction interval (5th–95th percentiles). For assessment of WWSN performance globally, we calculate the mean time to first detection, *T*_fd_, for each of the >3,200 subpopulations in the model (Fig. 1c). A notable aspect is the important spatial variability of *T*_fd_ based on the epidemic’s origin. For certain locations in Central Africa, *T*_fd_ is in the order of 100 days, while for many places in Europe, 15–25 days is more typical. While Fig. 2 shows that detection of epidemics emerging from some continents takes, on average, more time than for others, we also note an important heterogeneity within continents (Extended Data Table 1). For instance, in Africa, the 90% PI of *T*_fd_ ranges from 23 to 71 days. Zooming in at the level of statistical subregions, as defined by the United Nations geoscheme (Supplementary Fig. 6), we still find broad distributions of *T*_fd_ for all subregions. Middle Africa, for instance, is very dispersed, with 90% PI ranging from 28.2 to 84.5 days. This result indicates that, across regions and scales, there exist blind spots where detection of epidemics would take much longer if they were the source. Blind spots in the WWSN are partly due to low per-capita travel volume, as shown by the strong inverse correlation between international travel volume and *T*_fd_ (Supplementary Fig. 8). However, in some cases, detection at sentinel sites relies on importations from secondary outbreak locations with community transmission. This indirect path to reaching a sentinel further increases detection time from specific locations.

In Figs. 1 and 2, we assume that *p*_det_ in the WWSN is 16% and is uniform across all 20 sentinels. This probability of detection amounts to sampling from about 50% of international inbound flights, depending on the estimates for lavatory use and detectable shedding in fecal matter (Methods). In Supplementary Fig. 4, we report additional results for *p*_det_ as low as 4%, thus assuming a fraction of flights sampled in the range 12–25%. The aforementioned heterogeneity of *T*_fd_ persists across the full range of *p*_det_.

**Fig. 3:**
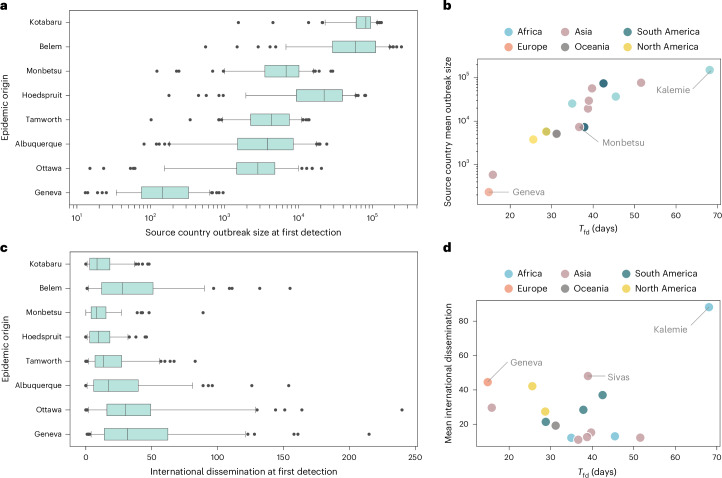
Additional performance metrics for global surveillance network at airports. **a**–**d**, We use the same baseline WWSN and disease parameters as in Fig. 1. GLEAM simulations (*n* = 100 for each origin) quantify the outbreak size—number of infectious individuals—in the country of origin (**a**,**b**) and the number of latent or infectious carriers disseminated internationally (**c**,**d**) at the time of first detection. **a**, Source country outbreak size at first detection. **b**, Source country mean outbreak size against *T*_fd_. Pearson correlation coefficient is 0.906 (90% CI 0.781–0.961, two-sided *P* = 1.4 × 10^−6^, testing noncorrelation) between *T*_fd_ and the logarithm of mean outbreak size in the country of origin (*n* = 16 origins). **c**, International dissemination at first detection. **d**, Mean international dissemination against *T*_fd_. Pearson correlation coefficient is 0.289 (90% CI −0.156 to 0.638, two-sided *P* = 0.277, testing noncorrelation) between *T*_fd_ and mean number of disease carriers disseminated internationally (n = 16 origins). **a**,**c**, Simulation results (*n* = 100) for eight origins, ordered by decreasing *T*_fd_. Boxplots show the median (center line), interquartile range (box), 90% central PI (whiskers, 5th–95th percentile) and outliers (black dots). Source data

While our analysis focuses on *T*_fd_ for situational awareness, the effectiveness of an outbreak response also depends on both outbreak size and the number of infections already dispersed internationally, along with their potential for cryptic transmission. To address these factors, we provide in Fig. 3 modeling estimates of both the number of infectious individuals at the source and the number of internationally dispersed infections at the time of first detection by the global WWSN (Supplementary Information 3). In Fig. 3a,b, it is evident that a longer *T*_fd_ is strongly associated with a larger outbreak in the country of origin (Pearson’s *r* = 0.906, two-sided *P* = 1.4 × 10^−6^). It is worth noting that our analysis assumes unmitigated scenarios until detection, although large outbreaks would probably be identified earlier at the source, triggering mitigation policies. Interestingly, the number of international introductions at the time of first detection remains relatively stable, typically within the range of a few dozen infected individuals (Fig. 3c,d). This quantity does not exhibit a statistically significant association with *T*_fd_ (Pearson’s *r* = 0.289, two-sided *P* = 0.277). Regardless of the outbreak’s origin, the WWSN detects the pathogen after a similar number of infections have spread internationally. This suggests that a global WWSN would provide early situational awareness for international public health responses, despite variability in detection time and local outbreak size.

### Effects of pathogen characteristics on the WWSN

**Fig. 4:**
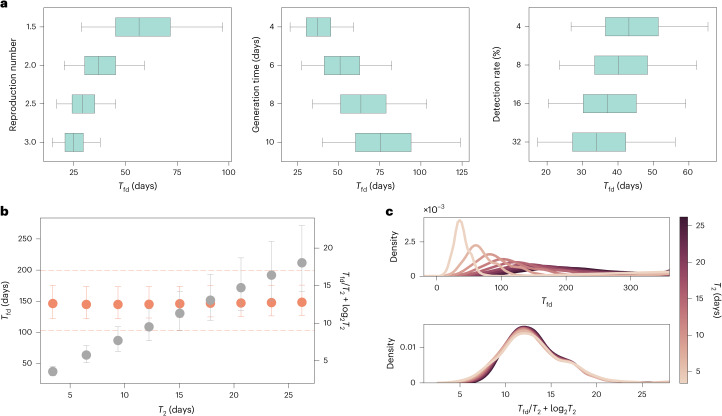
Changing transmission dynamics predictably affects *T*_fd_. **a**–**c**, We use the same baseline WWSN and detectable period as in Fig. 1. Unless specified, we maintain an average $\mathcal{R}_0$ of 2, *T*_gen_ of 4 days and 16% *p*_det_ at sentinels. All prediction intervals are obtained with *n* = 3,244 subpopulations. **a**, *T*_fd_ from all origins, with varying $\mathcal{R}_0$ (left), *T*_gen_ (middle) and *p*_det_ (right). Boxplots show the median (center line), interquartile range (box) and 90% central PI (whiskers, 5th–95th percentile) (*n* = 3,244); outliers outside the interval are not shown. **b**,**c**, We vary *T*_gen_ between 4 and 36 days, resulting in *T*_2_ between 3.4 and 26.2 days. **b**, *T*_fd_ and *T*_fd_/*T*_2_ + log_2_*T*_2_ as a function of *T*_2_. Circles indicate the median and error bars cover the interquartile range (*n* = 3,244). Dashed lines are purely a visual guide. **c**, Distributions of *T*_fd_ and *T*_fd_/*T*_2_ + log_2_*T*_2_ over all origins for different *T*_2_. For enhanced visualization, we use kernel density estimates for distributions. Source data

Although the model is designed to accommodate any specific pathogen and its shedding mechanisms, it is important to note that the natural history of a disease, particularly its key characteristic times and $\mathcal{R}_0$, considerably impacts *T*_fd_. In Fig. 4a, we show how the global distribution of *T*_fd_, aggregated over all locations, changes with variation in $\mathcal{R}_0$, generation time (*T*_gen_) and surveillance *p*_det_. A higher $\mathcal{R}_0$ and shorter *T*_gen_ lead to shorter *T*_fd_, and vice versa; the lower the probability of detection, the longer is *T*_fd_, although with limited impact. This can be explained by the exponential growth of epidemics in their early stages. The WWSN will typically start detecting cases when there is a sufficient number of detectable individuals (*D*) traveling through it; this number is approximately $D\propto {2}^{{T}_{{\rm{fd}}}/{T}_{2}}$, where *T*_2_ is the doubling time of the epidemic (here measured in days). Adjusting either $\mathcal{R}_0$ or *T*_gen_ greatly affects *T*_fd_, due to the change in *T*_2_. Conversely, changes in *p*_det_ do not similarly impact timing. Indeed, a twofold reduction in *p*_det_ implies a twofold increase in *D* before detection. However, this increase in *D* happens within the span of a single *T*_2_. The exponential growth also implies that the ratio *T*_fd_/*T*_2_ should be approximately constant as *T*_2_ of the epidemic varies. More precisely, as shown in Fig. 4b, the complete invariant quantity reads as

1 $${T}_{{\rm{fd}}}/{T}_{2}+{\log }_{2}{T}_{2}={\rm{constant}}.$$

The correction term log_2_*T*_2_ is necessary to account for the stochastic nature of the detection process^30^ (Supplementary Information 2). In Fig. 4c, we also show how the distributions of *T*_fd_ collapse onto one another when considering the invariant quantity in equation (1). In practical terms, altering the disease characteristics effectively results in a linear transformation of *T*_fd_ across all locations (Supplementary Fig. 10). Therefore, focusing on a specific parametrization does not result in any loss of generality of the results, allowing for consistent and generalizable analyses. Other aspects of disease transmission affecting *T*_fd_—overdispersion of the secondary infection distribution, length of the detectable period and seasonal change in the air travel network—have a more limited impact (Supplementary Table 4).

### Scaling and optimization of WWSNs

Both the number and geographic placement of sentinel airports are critical for optimization of WWSN effectiveness, representing a classic resource-constrained optimization challenge. For systematic assessment of network efficiency, we define more precisely *T*_fd_ ($\mathcal{S}$, *l*) as the mean time to first detection for a WWSN configuration, where $\mathcal{S}$ denotes the set of sentinel sites and *l* indicates the subpopulation at the epidemic’s origin. We can then average this metric over multiple origins by weighing each location according to a prior distribution, *P*(*l*), for the occurrence of an outbreak, resulting in

2 $${T}_{{\rm{fd}}}({\mathcal{S}})=\sum _{l}P(l)\,{T}_{{\rm{fd}}}({\mathcal{S}},l).$$

While *T*_fd_($\mathcal{S}$) is a well-defined indicator of performance, its value is sensitive to variation in disease transmission characteristics (equation (1)). To provide a more informative measure of network efficiency, we compare *T*_fd_($\mathcal{S}$) with *T*_fd_($\mathcal{C}$), where the latter is for a hypothetical complete WWSN $\mathcal{C}$ that includes all international airports globally. This comparison helps us quantify the relative performance of a specific sentinel configuration $\mathcal{S}$. We define excess time for the sentinel system $\mathcal{S}$ using the following formula:

3 $$E({\mathcal{S}})=100\times \frac{{T}_{{\rm{fd}}}({\mathcal{S}})-{T}_{{\rm{fd}}}({\mathcal{C}})}{{T}_{{\rm{fd}}}({\mathcal{C}})}.$$

This metric represents the additional percentage of time required for the system $\mathcal{S}$ to achieve its first detection compared with the complete network.

**Fig. 5:**
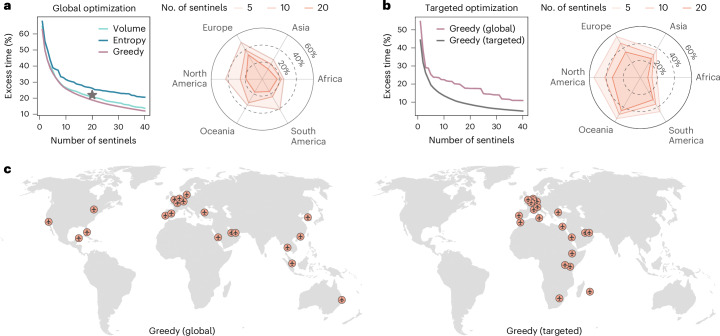
Scaling and optimization of a global surveillance network at airports. **a**–**c**, Using the same disease parameters as in Fig. 1, we evaluate *T*_fd_ and compute excess time relative to the complete WWSN, varying the number of sentinel airports and selection strategies. **a**, Global optimization assumes that all subpopulations are equally probable epidemic origins, with the star indicating the baseline network’s excess time. **b**, Targeted optimization minimizes excess time for epidemics originating in Africa. **a**,**b**, Radar charts show excess time by continent for global and targeted greedy strategies. A balanced strategy minimizes excess time across all regions, while lower excess times for a specific region reflect targeted optimization. **c**, Spatial distribution of the first 20 sentinels under global and targeted optimization strategies. Source data

We use three different strategies to define the geographic distribution of the sentinel network: (1) ranking of airports based on their international inbound passenger volume^11^; (2) ranking airports by their entropy in traffic flows—a measure of diversity that favors airports offering wide geographical connectivity; and (3) using a greedy optimization strategy that minimizes *T*_fd_ (Methods). We acknowledge here that a wide range of approaches and alternative optimization strategies for network surveillance can be explored^31–33^. However, evaluation of these algorithms is beyond the scope of this manuscript and is left for future case-specific studies. In Fig. 5a we show excess time for the three different strategies considered, assuming a homogeneous prior for the source of an epidemic, irrespective of the area or population size (that is, *P*(*l*) = constant for all *l*). While the greedy approach systematically provides the lowest excess time, all three strategies show similar performance despite different network configurations (Supplementary Table 6). The radar chart also shows that the greedy strategy achieves relatively balanced geographical surveillance compared with the complete WWSN. Most notably, optimization analysis yields diminishing returns as the number of sentinels increases. A network of 20 sentinels detects outbreaks only ~20% more slowly than a system with thousands of airports, and doubling this number improves detection time by less than 10%. This result indicates a highly cost-effective trade-off between the efficiency of the WWSN and the resources allocated to it. A small number of sentinels provides near optimal efficiency.

Some diseases are endemic in only certain parts of the world, or have clear seasonal patterns; besides, we have shown that *T*_fd_ is higher for some geographical areas than others (Fig. 2). For these reasons, the WWSN can be adapted by biasing the optimization procedure to improve detection capabilities for specific geographical areas. The greedy optimization approach allows for this by adjusting the prior function *P*(*l*). For instance, to minimize *T*_fd_ for outbreaks originating in Africa, we can set *P*(*l*) = constant if *l* is in Africa and *P*(*l*) = 0 otherwise. Figure 5b compares excess time between conventional global greedy optimization and our targeted greedy optimization strategy. The radar chart highlights the bias introduced by targeted optimization, with coverage favoring Africa at the expense of other regions. Figure 5c shows how sentinel geographical placement shifts substantially when optimization focuses on a specific area. Targeting Africa leads to a higher concentration of sentinels in Africa and Europe, reflecting traffic flow patterns. European hubs are selected due to their high international travel volume, including traffic from many African countries. For example, Paris Charles de Gaulle Airport emerges as the second-top sentinel when optimization is targeting the African continent (Supplementary Table 6). Sentinel selection, however, depends on the specific disease considered and regional characteristics, requiring case-by-case optimization. These findings pave the way for dynamic adaptation of the WWSN to evolving epidemic knowledge and geographic spread.

### Situational awareness with WWSNs

**Fig. 6:**
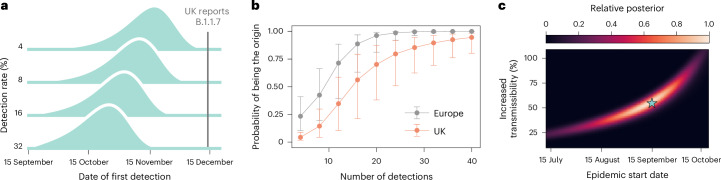
WWSN as an early warning system with inferential capabilities. **a**–**c**, We simulate a counterfactual scenario of the emergence of the SARS-CoV-2 Alpha variant with a global WWSN in place, using the baseline surveillance system from Fig. 1. The wild strain (effective reproduction number ${{\mathcal{R}}}_{{\rm{eff}}}^{{\rm{ws}}}=1.1$) and Alpha variant (${{\mathcal{R}}}_{{\rm{eff}}}^{{\rm{alpha}}}=1.7$, 55% increase in transmissibility) are modeled with a *T*_gen_ of 6.5 days and postinfectious period of 10 days. The outbreak starts with 20 infectious and 20 latent individuals in London and southeast England on 15 September, 2020. **a**, Distribution for the date of first infection for varying *p*_det_. **b**,**c**, Inference using GLEAM-generated data with 16% *p*_det_. **b**, Geolocation of the source improves as detections accumulate; posterior distributions for epidemic origin are based on sentinel detection counts, with markers for median posterior values and error bars for interquartile range (derived from 1,250 detection time series). **c**, Joint posterior distribution of Alpha’s increased transmissibility and epidemic start date, averaged over 125 detection time series. Blue star represents ground truth (15 September and 55% increased transmissibility). The use of Gouraud interpolation enhances visualization. Source data

WWSNs can be used to provide evolving situational awareness on emerging infectious disease threats. To illustrate the potential use of WWSNs in gathering epidemiological information, we explore the emergence of the SARS-CoV-2 Alpha variant (B.1.1.7) in Fall 2020 (Supplementary Information 4)^34–36^. More precisely, we consider a hypothetical scenario where the baseline WWSN illustrated in Fig. 1 is assumed to be operational. The study uses air travel data from September to November 2020, and in Fig. 6a we present probable distributions for the date of first detection of the Alpha variant. Our findings show that, even with 4% *p*_det_, the Alpha variant would probably have been detected by November, with a median detection date of 13 November and 90% PI from 15 October to 1 December. At 16% *p*_det_, the first detections are projected by late October, with a median date of 29 October and 90% PI from 2 October to 16 November. Because the Alpha variant was first reported by the UK government on 14 December 2020 (ref. ^37^), these results show the potential of a global WWSN as an effective early warning system.

Alongside tracking of the initial international spread, the WWSN can also deliver timely information on the origin of an outbreak and help in understanding its growth dynamics. In Fig. 6b, we show the probability that the WWSN correctly identifies the continent and country of origin as multiple detections accumulate in the system. This is achieved by calculating the posterior distribution *P*(*l|***d**) for each subpopulation *l* as being the origin of an epidemic based on the cumulative number of detections at each sentinel ${{{\mathbf{d}}}}={({d}_{\nu })}_{\nu \in {\mathcal{S}}}$ (Supplementary Information 4). Our analysis suggests that the source country could have been accurately identified after about 20 detections, probably by 5 December in over 50% of model realizations, with 16% *p*_det_. In practice, a more efficient adaptive strategy could involve targeted sampling of aircraft from regions suspected as the outbreak’s epicenter, enabling quicker and more precise source identification. In addition, multiple detection events can be utilized to estimate key epidemic parameters, including growth rate, onset time and reproduction number, given knowledge of the contagion’s *T*_gen_. Here, we focus on inferring the epidemic start date and the Alpha variant’s increased transmissibility compared with the original SARS-CoV-2 strain. As of 14 December 2020, when the UK first reported the Alpha variant, detection events at sentinel sites produce a joint posterior distribution for the epidemic start date (90% credible interval (CI) 29 July–12 October) and increased transmissibility (90% CI, 25–91%), as shown in Fig. 6c. The high-posterior-density region also matches closely the value of the simulation experiment—that is, a start date of 15 September and 55% increased transmissibility. The detailed inference procedure and individual posterior distributions for different time series are reported in Supplementary Information 4. Additional evidence of the timely situational awareness capacities provided by a global WWSN is presented in Supplementary Information 4, with the hypothetical scenario in which a global WWSN would have been operational at the time of the emergence of SARS-CoV-2 in Wuhan, China.

## Discussion

Our findings demonstrate the potentially important role of WWSNs in shortening pathogen detection time, overcoming some of the challenges faced with standard symptoms-based passenger screening across regions^38^. Gaining even a few extra days of situational awareness about a pathogen’s introduction can be critical for outbreak control. For example, in the case of the Alpha SARS-CoV-2 variant, sentinel systems can retrospectively determine the pathogen’s introduction date and geographic spread, informing travel restrictions and border screening policies. While these measures are costly, they are often implemented too narrowly or too late to be fully effective. WWSNs can provide timely and precise surveillance data, supporting more effective public health responses. In addition, our framework identifies potential blind spots in WWSNs, guiding the integration of complementary surveillance methods, such as community wastewater monitoring, to increase the network’s coverage and effectiveness^39^.

The strategies and numerical experiments presented here aim to present the capabilities of a WWSN rather than focus on a specific disease or outbreak. The proposed model integrates real-world airline data and can be extended to include travel disruptions (for example, cancellations, rerouting or restrictions)^26^. It can also account for vaccination effects, such as reduced transmissibility, immune proportions and varying viral shedding rates among vaccinated individuals. Future studies could expand the model’s application by incorporating knowledge and experiences from the surveillance of specific pathogens, such as arboviruses and influenza^40,41^. In addition, incorporation of factors influencing zoonotic spillovers—shaped by socioeconomic, environmental and ecological dynamics—will enhance our understanding of emerging diseases and our predictive capabilities^42–45^.

Future modeling efforts should incorporate the logistical capabilities of WWSNs, exploring operational implementations such as rotating testing schedules and cadences across sentinel sites to address logistical constraints. For instance, although we used the same detection probability across the WWSN, in Supplementary Information 2 we present a sensitivity analysis with varying levels of heterogeneity for detection probability at sentinel airports, demonstrating that *T*_fd_ remains robust, with results similar to the homogeneous case shown in Fig. 1. Our modeling framework also enables targeted and adaptive strategies to enhance pathogen screening efficiency while optimizing resource use. It is worth remarking, however, that following detection of an outbreak, response actions must balance social, economic and public health priorities while considering available resources, logistical constraints and the specific disease threat. Our modeling framework supports this process by planning of in silico WWSNs that aligns with the potential response strategies.

Like all modeling studies, our analysis contains assumptions and limitations that must be clearly identified. We model air travel as an independent process for individuals, neglecting clusters and household travel. Spillover events affecting isolated individuals with disease-specific behaviors may lead to variable early-stage dynamics; we mitigate these effects by using small clusters of infected individuals as initial conditions. More technically, some of our analytics rely on a multitype branching process that ignores saturation effects from finite populations. While these effects are minor and do not impact early outbreak conclusions, they should be considered when analyzing WWSN performance for large epidemics or endemic situations. In addition, the model does not account for false positives or positive tests resulting from uncleaned wastewater tanks between flights^19^. While this is unlikely to affect considerably our analysis of *T*_fd_, it may influence analysis of the situational awareness capabilities of the WWSN. Future studies should incorporate test specificity, and potential wastewater tank cross-contamination, into the model. This will be crucial for decision-making, particularly when detecting rare but high-consequence pathogens.

Taking into account its limitations, our study provides a general framework for modeling wastewater surveillance at airports, supporting public health decision-making through both planning and surveillance modes. In the planning mode, the model identifies optimal sentinel networks and evaluates effectiveness using metrics such as *T*_fd_. In surveillance mode, it estimates key epidemiological parameters such as transmission characteristics and outbreak timing from detection events. Furthermore, although our study focuses on aircraft wastewater surveillance, it can also be applied to environmental monitoring and other travel-based surveillance methods, such as nasal swab testing, offering a comprehensive modeling platform for genomic and travel-based disease surveillance.

## Methods

### GLEAM

GLEAM is a computational platform used for modeling epidemic spread, combining stochastic elements and spatial data in an age-structured, metapopulation framework^21–23^. GLEAM divides the world into distinct geographic subpopulations using a Voronoi tessellation of the Earth’s surface, with each subpopulation centered around major transportation hubs such as airports. These subpopulations are detailed with high-resolution data about population demographics, age-specific contact patterns, health infrastructure and other relevant attributes based on available data. GLEAM incorporates a human mobility layer into its modeling, using data from various sources, including the OAG and International Air Transport Association (IATA) databases. This layer includes both short-range (for example, commuting) and long-range (for example, flights) mobility data, and creates a network of daily passenger flows between airports worldwide. The model uses a worldwide homogeneous standard for commuting and compensates for missing information with synthetic data based on the ‘gravity law’ calibrated with real data^21,23^.

GLEAM tracks the number of individuals in each disease state for all subpopulations over time. It simulates travelers’ movements through the flight network, with air travel probabilities varying by age group. Finally, the disease dynamics and the detection process at airports within GLEAM are simulated using stochastic binomial chain processes. These processes rely on parameter values sourced from existing literature, defining the natural history of the infection being modeled. See Supplementary Information 1 for a more technical description of the model. All our analyses make use of a global air travel network capturing the period September 2022 to August 2023, except for the case studies on the emergence of COVID-19 (Supplementary Section 4) and the SARS-CoV-2 Alpha variant, for which we use data from December 2018 to February 2019 (the available air travel networks at the beginning of the COVID-19 pandemic) and September to November 2020, respectively.

### Disease progression and transmission dynamics

For modeling of disease transmission within subpopulations and detections at airports following air travel, we make use of a standard compartmentalization scheme for disease progression. Each individual, at any time point, is assigned to a compartment corresponding to their particular disease-related state. An individual who becomes infected will go through the following sequence of states: susceptible (S, pre-exposure), latent (L, exposed, but does not yet transmit the infectious pathogen), infectious (I, can transmit the disease), postinfectious (P, no longer infectious) and recovered (R). In our model, we assume that only infectious and postinfectious can be detected through wastewater, which we regroup under the detectable (D) state. Inclusion of the postinfectious state in our model is necessary because viruses such as SARS-CoV-2 remain detectable in wastewater well beyond the active infectious period of the single individual^6,28,29^. Furthermore, since the period an individual spends in a certain compartment is typically not exponentially distributed^46,47^, we add realism to our model by decomposing the infectious and postinfectious states into two substates, namely I_1_ and I_2_, and P_1_ and P_2_. Parameters and details on the contagion dynamics (*T*_gen_, *T*_2_, detectable period and $\mathcal{R}_0$) are reported in the Supplementary Information.

### Aircraft wastewater detection

In our model, a detectable individual passing through a sentinel site is detected with probability *p*_det_ that depends on several factors, including the cadence and sampling of airport wastewater surveillance, the duration of the flight, the diverse sociodemographic profiles of the passengers^12^ and so on. In our analysis we assume that, on average, *p*_det_ is uniform across all inbound international flights arriving at any given sentinel site. To provide a rationale for the spectrum of *p*_det_ examined in this study, we break down probability into the following components—*p*_det_ = *p*_lav_ × *p*_shed_ × *p*_sample_, where *p*_lav_ represents the likelihood that an individual will utilize the lavatory and consequently deposit detectable genetic traces of the pathogen in the wastewater; *p*_shed_ denotes the probability that a detectable individual is actively shedding the pathogen at levels sufficient for detection in the wastewater; and *p*_sample_ refers to the proportion of flights that are subjected to sampling at the sentinel airport.

The proportion of adult passengers defecating on flights, critical for estimation of *p*_lav_, is surveyed to be less than 13% on short-haul and less than 36% on long-haul flights^12^. Further, *p*_shed_, the probability of detectable pathogen shedding in fecal matter, ranges between 30 and 60% for SARS-CoV-2 (ref. ^12^). This would correspond to a *p*_det_ per passenger on sampled flights (*p*_lav_ × *p*_shed_) in the range 11–22% on long-haul flights. These estimates are possibly a large underestimation for viruses such as SARS-CoV-2, because individuals can leave genetic material in the wastewater without defecating^48^, such as by disposing of a used tissue or spitting in the toilet. Indeed, previous studies^19^ have shown 83.7% accuracy in detection of COVID-19 on repatriation flights using wastewater analysis. Translating this value to an individual’s marginal detection probability is complex, because the number of COVID-19 cases per flight varied considerably, averaging 4.62 cases. Accounting for false-positive wastewater results, assuming each case had an equal probability to be detected and a flat prior, we find a median marginal *p*_det_ of 51% (90% CI, 28–72) on sampled flights. However, this value could be inflated, notably due to the persistent nature of fecal RNA shedding compared with respiratory shedding^49^, and accounting for the fact that not all international flights are long haul.

Interpolating from the above values, we consider an estimate of 32% for the *p*_det_ of a single detectable passenger on a flight subject to wastewater sampling (*p*_lav_ × *p*_shed_). In our analysis, we assume a baseline *p*_det_ of 16%, which corresponds to sampling 50% of international flights (*p*_sample_ = 50%). Given the variability in these estimates, we also explore a range of *p*_det_, from as high as 32% to as low as 4%, acknowledging that only a small fraction (for example, 12%) of flights might be sampled. Our sensitivity analysis presents findings across this full spectrum of *p*_det_ (Figs. 4a and 6a and Supplementary Fig. 4).

### PGF analytics

The mechanistic GLEAM model uses large-scale stochastic simulations that are computationally intensive. To streamline our analysis, for most of the results in this paper we utilize PGFs to efficiently extract the required analytic information from the data and model. PGFs are a standard tool in mathematical epidemiology^50,51^ and have found many applications, including the quantitative analysis of the risk of disease introduction^52–55^.

PGFs are useful in counting elements. Here we are counting individuals based on certain properties: their age, their location and their epidemiological state. We define *s*_*σ*_ as the number of individuals of type *σ*. For instance, *s*_*σ*_ could represent the current number of latent individuals in a given location and of a certain age. We use the vector **s** to encapsulate all these numbers.

To capture the full stochastic evolution of the system, we encode the probability distribution *P*(**s**, *t*) with a multivariate PGF

4 $${\Psi }^{t}({\boldsymbol{x}})=\sum _{{{{\mathbf{s}}}}}P({{{\mathbf{s}}}},t)\prod _{\sigma }{x}_{\sigma }^{{{s}}_{\sigma }},$$

where the sum (product) runs over all possible values of **s** (*σ*) and each *x*_*σ*_ is a variable that acts as a placeholder to encode probability distribution. The vector **x** encapsulates all these variables.

In the early stage, a structured metapopulation epidemic model such as GLEAM can be described by a multitype branching process^56^, in which case we solve the PGF through the recursive equation

5 $${\Psi }^{t+1}({\boldsymbol{x}})={\Psi }^{t}[{{{\mathbf{F}}}}({{{\mathbf{x}}}})],$$

where **F**(**x**) is a vector and each element *F*_*σ*_(**x**) is itself a PGF that characterizes the offspring distribution of an individual of type *σ*. Computing the full distribution *P*(**s**, *t*) is out of reach—the number of terms explodes combinatorially. However, computing marginal or joint distributions for a few observables, such as the total number of individuals in a particular state, is possible (Supplementary Information 1).

Taken together, the recursive evaluation of PGFs and their numerical inversion to recover probability distributions represents a very efficient computational alternative to Monte Carlo simulations. This crucially allows us to extract distributions of observables, like the time to first detection, assuming that the epidemic could have started from any of the >3,200 subpopulations of our model, a task that would be prohibitive with a purely simulation-based framework. See Supplementary Information 1 for an in-depth description and characterization of the PGF methodology.

### WWSN optimization algorithms

The heuristic optimization of global WWSNs selects sentinel sites based on their rankings according to the following measures. Let *N*_*l*→*ν*_ be the number of individuals per day who will travel and arrive at airport *ν* on an international flight, either as a final destination or for a connection; the flows of international passengers generate a weighted bipartite network connecting international airports *ν* to subpopulations *l*. We can therefore rank airports based on their volume of international travel:

6 $${c}_{\nu }^{{\rm{vol}}}=\sum _{l}{N}_{l\to \nu }.$$

A second-ranking measure is based on each airport traffic entropy, defined as:

7 $${c}_{\nu }^{{\rm{ent}}}=-\sum _{l}\left(\frac{{N}_{l\to \nu }}{{\sum }_{{l}^{{\prime} }}{N}_{{l}^{{\prime} }\to \nu }}\right)\log \left(\frac{{N}_{l\to \nu }}{{\sum }_{{l}^{{\prime} }}{N}_{{l}^{{\prime} }\to \nu }}\right).$$

This expression is also known as Shannon’s diversity index; this measure favors airports with a broad and homogeneous coverage of the different subpopulations.

A more refined optimization algorithm aims at minimizing the *T*_fd_ of an epidemic, averaged over all potential origins. We can assign an arbitrary prior probability *P*(*l*) for location *l* to be the origin of an epidemic, resulting in the following objective function:

8 $$\Phi ({\mathcal{S}})\equiv {T}_{{\rm{fd}}}({\mathcal{S}})=\sum _{l}P(l)\,{T}_{{\rm{fd}}}({\mathcal{S}},l),$$

where *T*_fd_($\mathcal{S}$, *l*) is *T*_fd_, assuming that the epidemic started in subpopulation *l* and that the WWSN consists of the set of sentinel airports $\mathcal{S}$. For global optimization we use $P(l)={\rm{const}}.\,\forall l$ —that is, all subpopulations are an equiprobable source. For targeted optimization, we use *P*(*l*) = const. for locations in the targeted region and *P*(*l*) = 0 otherwise.

We conjecture that $-\Phi ({\mathcal{S}})$ is a monotone submodular set function^57^. We prove this statement in Supplementary Information 2 for a very accurate approximation of $-\Phi ({\mathcal{S}})$, but the exact case remains to be proven. Monotone submodular functions have desirable properties in relation to discrete optimization problems: we have a guarantee on the performance of a greedy optimization algorithm—that is, there exists an upper bound on the value of $\Phi ({\mathcal{S}})$ obtained through this approach^57^. Most importantly, in practice, a greedy algorithm should find a solution that is very close to the optimal one. Consequently, to minimize the objective function, equation (8), we use the following greedy optimization scheme:

1. Define an initial set *S* (can be empty).
2. For each airport $\nu \notin {\mathcal{S}}$, compute $\Phi ({\mathcal{S}}\cup \{\nu \})$.
3. Update the set ${\mathcal{S}}\leftarrow {\mathcal{S}}\cup \{{\nu }^{\star }\}$, where *ν*^⋆^ is the sentinel airport that minimizes the objective function.
4. Repeat steps 2 and 3 until a desired number of sentinels is reached.

### Reporting summary

Further information on research design is available in the Nature Portfolio Reporting Summary linked to this article.

## Online content

Any methods, additional references, Nature Portfolio reporting summaries, source data, extended data, supplementary information, acknowledgements, peer review information; details of author contributions and competing interests; and statements of data and code availability are available at 10.1038/s41591-025-03501-4.

## Supplementary information

Supplementary Information Supplementary Sections 1–5, Figs. 1–21, Tables 1–6 and References.

Reporting Summary

## Source data

Source Data Fig. 1 Statistical source data.

Source Data Fig. 2 Statistical source data.

Source Data Fig. 3 Statistical source data.

Source Data Fig. 4 Statistical source data.

Source Data Fig. 5 Statistical source data.

Source Data Fig. 6 Statistical source data.

## Figures and Tables

**Extended Data Table 1:**
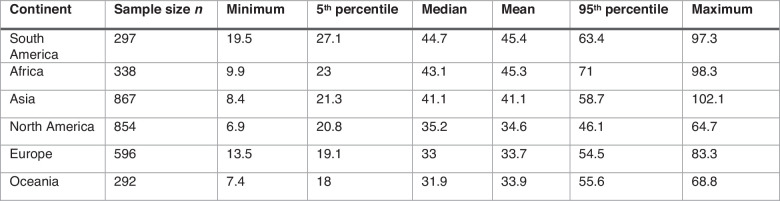
Statistics of the mean time to first detection

Numerical values for some of the statistics of the mean time to first detection presented in Fig. 2.

## Data Availability

Proprietary airline data are commercially available from OAG (https://www.oag.com/passenger-booking-data). Data on the effective reproduction number in London and southeast England during fall 2020 are available from the UK government website (https://www.gov.uk/guidance/the-r-value-and-growth-rate). Source data are provided with this paper.

## Code availability

The mechanistic GLEAM model is publicly available at http://www.gleamviz.org/ and can be used to generate simulation data. The code associated with PGF methodology is publicly available on GitHub (https://github.com/mobs-lab/pgfgleam), with documentation to compute the analytics in this paper. All data analyses of model results were performed using Python v.3.10.8.
