# Supplementary material for: Pandemic monitoring with global aircraft-based wastewater surveillance networks
Source: Nat Med. 2025 Feb 12;31(3):788–96. doi: 10.1038/s41591-025-03501-4 (PMC11922747; doi:10.1038/s41591-025-03501-4)
Supplement: Supplementary file 1 — Supplementary Sections 1–5, Figs. 1–21, Tables 1–6 and References. [file 41591_2025_3501_MOESM1_ESM.pdf]

nature medicine

Supplementary information https://doi.org/10.1038/s41591-025-03501-4

Pandemic monitoring with global aircraft-based wastewater surveillance networks

In the format provided by the authors and unedited

Pandemic monitoring with global aircraft-based wastewater surveillance networks

Supplementary Information

Guillaume St-Onge[1,2,3], Jessica T. Davis[1,3], Laurent Hébert-Dufresne[4,5,6], Antoine Allard[4,6,7], Alessandra Urbinati[1,3], Samuel V. Scarpino[2,3,4,8,9], Matteo Chinazzi[1,2,3], and Alessandro Vespignani[1,2,3,10]

[1] *Laboratory for the Modeling of Biological and Socio-technical Systems, Northeastern University, Boston, MA 02115, USA*
[2] *The Roux Institute, Northeastern University, Portland, ME 04101, USA*
[3] *Network Science Institute, Northeastern University, Boston, MA 02115, USA*
[4] *Vermont Complex Systems Institute, University of Vermont, Burlington, VT 05405, USA*
[5] *Department of Computer Science, University of Vermont, Burlington VT 05405, USA*
[6] *Département de physique, de génie physique et d'optique, Université Laval, Québec City, QC G1V 0A6, Canada*
[7] *Centre interdisciplinaire en modélisation mathématique, Université Laval, Québec City, QC G1V 0A6, Canada*
[8] *Institute for Experiential AI, Northeastern University, Boston, MA 02115, USA*
[9] *Santa Fe Institute, Santa Fe, NM 87501, USA*
[10] *Institute for Scientific Interchange Foundation, Turin, 10126, Italy*

Contents

1 Model description **1**
1.1 Global Epidemic and Mobility Model 1
1.2 Probability generating function framework 4

2 Time to first detection for a global wastewater surveillance system at airports **10**
2.1 Variation with the disease natural history 11
2.2 Sensitivity analyses 13
2.3 Optimization of the time to first detection 15

3 Additional performance metrics **21**

4 Retrospective counterfactual scenarios **21**
4.1 SARS-CoV-2 Alpha variant emergence 21
4.2 SARS-CoV-2 (wild strain) emergence 25

5 Airport table for the sentinel surveillance system **28**

1 Model description

1.1 Global Epidemic and Mobility Model

The GLEAM (Global Epidemic and Mobility) model is a stochastic epidemic metapopulation framework that incorporates age-based contact matrices and data on human mobility. The approach has been documented in previous publications [1, 2] and has been used to study the spread of diseases such as Ebola [3], Zika [4], and COVID-19 [5, 6].

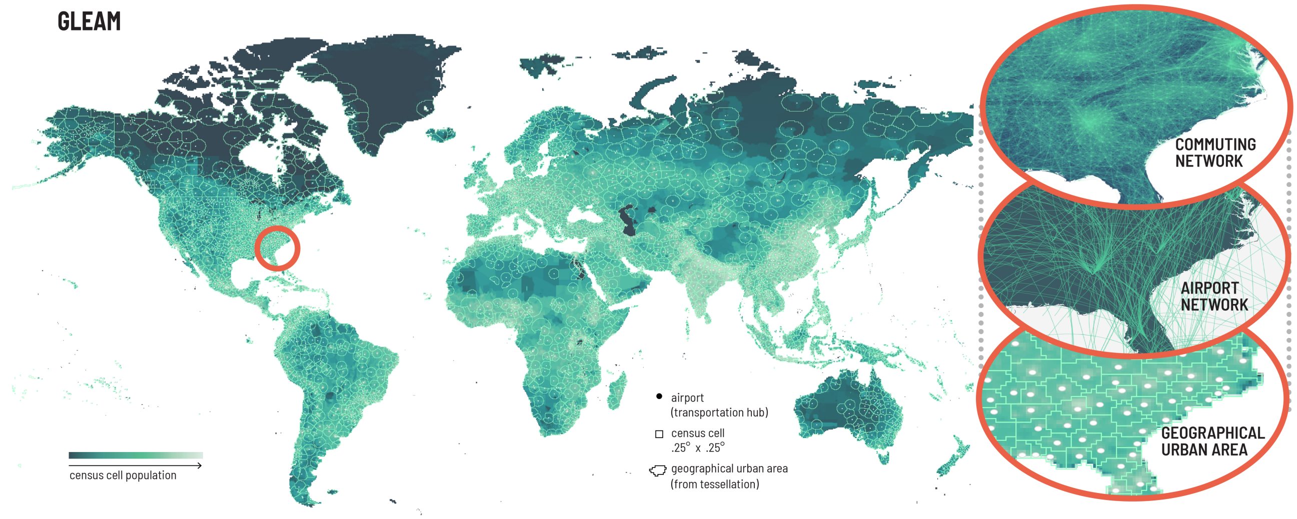

Figure S1: Schematic representation of GLEAM. (left) Voronoi tessellation of the globe centered around transportation hubs (airports) creating each of the 3200+ geographical units that we refer to as subpopulations. Each subpopulation is constructed from census cells of approximately 25km × 25km. (right) For a particular region, we illustrate the subpopulations and the two mobility layers— air travel (long-range) and commuting (short-range).

The model uses a Voronoi tessellation to create a metapopulation network with over 3,200 subpopulations, covering areas of the globe inhabited by humans. These subpopulations are anchored around major transport hubs like airports and are themselves divided into cells measuring around 25 x 25 kilometers, equivalent to 15 x 15 arc minutes. In addition, the model integrates cell-level population data [7] and subpopulation-level age-specific contact patterns using the contact matrices developed in [8]. Here, we consider individuals divided into 5 age groups: [0-4, 5-17, 18-49, 50-64, 65+].

The individual subpopulations are connected through a human mobility layer that combines both short-range (i.e., commuting) and long-range (i.e., flights) mobility data. Commuting data is sourced from the Offices of Statistics for 30 countries on 5 continents. To harmonize the varying spatial resolutions of commuting data across different countries and to address data availability gaps, the short-range mobility layer is synthetically generated where necessary. This is achieved by relying on the "gravity law" [1, 9, 10], which is calibrated on the available data. Air-travel data from the Official Aviation Guide (OAG) and IATA databases is used to build an origin-destination network, incorporating connecting flight information. The network provides daily passenger flows between airports globally, which we systematically map and aggregate at the subpopulation level. Figure S1 displays the geographical resolution of the model for selected regions, illustrating both the short-range and long-range mobility networks and the global population structure.

Compartmental model

The synthetic world created by the human mobility layer couples the epidemic dynamics unfolding within each subpopulation. To model the infection process, we adopt an extended SLIR-like model in which individuals are either susceptible (S), latent (L), infectious (I), post-infectious (P), or removed (R). We further subdivide the infectious and post-infectious compartments into two stages, namely I_1, I_2, P_1, and P_2 to more realistically model the timing of the disease progression [11, 12].

Susceptible individuals become latent through interactions with infectious individuals, at a rate Λ, the *effective*

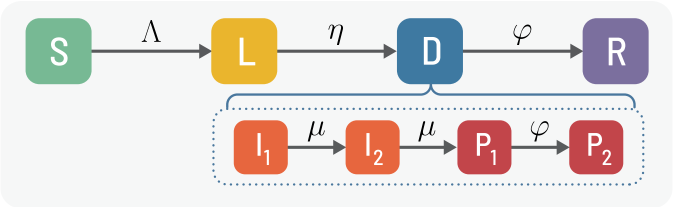

Figure S2: Compartmental model for wastewater surveillance at airports.

force of infection, which depends on the age of the susceptible individual but also on the whole state of the system, and thus varies in time. An important factor influencing the force of infection is the *basic reproduction number* $\mathcal{R}_0$—the average number of secondary infections caused by an infectious individual in a fully healthy population. Note that in our model, the reproduction number $\mathcal{R}_0$ will vary slightly from one subpopulation to another, since it is proportional to the largest eigenvalue of the age-structured contact matrices [8].

Latent individuals then progress to the first infectious stage at a constant rate inversely proportional to the mean latent period $\eta \equiv T_{\mathrm{lat}}$. Infectious individuals in the first stage I_1 progress to the second stage I_2 at a rate $\mu \equiv 2 \times T_{\mathrm{inf}}^{-1}$, where T_{inf} is the mean infectious period; the process is identical for the transition of I_2 individuals to P_1. Similarly, post-infectious individuals in the first stage (P_1) progress to the second stage P_2 at a rate $\varphi \equiv 2 \times T_{\mathrm{post}}^{-1}$, where T_{post} is the mean post-infectious period, then progress to the recovered stage at the same rate φ. The post-infectious period is the length of time that an individual can still shed the virus and remain detectable through wastewater, but not generate any more new infections. Once an individual is in the removed compartment, it can no longer be detected. Here we assume only infectious and post-infectious individuals can be detected through wastewater, which is why we regroup them in a *detectable* (D) meta-compartment. The various transitions and their rates are portrayed in Fig. S2.

Because of the subdivision of the infectious and post-infectious states, the infectious and post-infectious periods are gamma-distributed, while the latent period is exponentially distributed. The *generation time*, the time between the exposures of an infector-infectee pair, will have a mean T_{gen} expressed as [11, 12]

$$T_{\mathrm{gen}} = T_{\mathrm{lat}} + \left(\frac{n_{\mathrm{inf}}+1}{2n_{\mathrm{inf}}}\right) T_{\mathrm{inf}} \ , \tag{1}$$

where n_{inf} is the number of infectious states—in our model $n_{\mathrm{inf}} = 2$. Similarly, the *detectable period*—the length of time an individual can be detected through wastewater—has a mean of $T_{\mathrm{det}} = T_{\mathrm{inf}} + T_{\mathrm{post}}$.

In this study, we adjust the parameters of our compartmental model disease dynamics by fixing the reproduction number, mean generation time, and mean detectable period. In Table S1 we provide the parameters' ranges considered in this study and compare them with estimated ranges of SARS-CoV-2.

Stochastic simulation of the transmission and mobility dynamics

With the mobility data layers and the disease dynamics defined, the number of individuals in each compartment c, age bracket a, and subpopulation l follows a discrete and stochastic dynamical equation that reads as

$$X_l^{[c,a]}(t+\Delta t) - X_l^{[c,a]}(t) = \Delta X_l^{[c,a]} + \Omega_l([c,a]) \tag{2}$$

where the term $\Delta X_l^{[c,a]}$ represents the changes induced by the disease dynamics and $\Omega_l([c,a])$ represents the variations due to air travel. In this study, each day is subdivided in $m = 12$ time steps, i.e., $\Delta t = 1/m$ days. While the disease dynamics part $\Delta X_l^{[c,a]}$ is applied at every of these time steps, the variations due to traveling $\Omega_l([c,a])$ are introduced daily. The latter $\Omega_l([c,a])$ is directly extracted from a multinomial distribution associated with the age-specific probability of travel per day, as defined by the global air-travel network.

The variation $\Delta X_l^{[c,a]}$ is determined by summing over all transitions in and out of the disease compartment c for

Table S1: Range of disease parameters explored in this study compared with estimates for the early transmission of SARS-CoV-2 (wild strain). †Estimates combine mean generation time and serial interval.

Parameter	Range considered	SARS-CoV-2 (wild strain)	References
Reproduction number	[1.5, 3]	[2, 3.5]	[13–16]
Mean generation time	[4, 36] days	[4, 7.5] days†	[14, 17]
Mean detectable period	[7.7, 22.7] days	[7, 22] days	[18–20]

the age group a,

$$\Delta X_l^{[c,a]} = \sum_{[c',a]} \left\{ -\mathcal{D}_l([c,a],[c',a]) + \mathcal{D}_l([c',a],[c,a]) \right\}, \tag{3}$$

where $\mathcal{D}_l([c,a],[c',a])$ represents the number of transitions from $[c,a]$ to $[c',a]$ during the time interval Δt. For all spontaneous transitions, like from latent to infectious, $\mathcal{D}_l([c,a],[c',a])$ is simply extracted from a binomial distribution.

The generation of new infections is a more complex procedure. First, it hinges on the age-structured contacts matrix $\mathbf{C}$, which gives the expected number of contacts per day between each age pair (a, a') in a given location l. We consider interactions in four social settings: contacts at school ($\mathbf{C}_{school}$), workplace ($\mathbf{C}_{work}$), home ($\mathbf{C}_{home}$), and in the general community ($\mathbf{C}_{community}$), which are linearly combined to create $\mathbf{C}$, as defined in Ref. [8]. Second, the mobility due to the commuting flows is also taken into account using a time scale separation approximation, as detailed in Ref. [1]. Altogether, these two factors contribute to the effective force of infections $\Lambda([l,a])$ acting on susceptible individuals in subpopulation l and age a, resulting in new transitions of the form $\mathcal{D}_l([c,a],[c',a])$.

Initial conditions are established by defining the quantity and location of individuals who can spread the infection. Subsequently, GLEAM tracks the number of individuals in each disease compartment for every subpopulation over time. Please see Ref. [2] for a more in-depth discussion of the simulation framework.

Wastewater detection at airports

We assume that a subset of all airports $\mathcal{S} = \{\nu_1, \nu_2, \dots\}$—*sentinels*—monitor the wastewater of incoming *international* aircrafts. Consequently, each detectable international traveler passing through a sentinel has a probability p_{det} of leading to a detection. As detailed in the Methods section of the main text, the probability p_{det} is a complex quantity combining multiple factors, such as sampling frequency of aircraft, length of the flight, lavatory usage estimates, etc. We settled on performing an extensive sensitivity analysis spanning the range 4–32%.

In GLEAM's stochastic simulation process, detections at sentinels are aggregated during the post-processing of the simulation data, which keeps track of all mobility-induced changes $\Omega_l([c,a])$. Since GLEAM produces origin-destination travel patterns at the level of subpopulations l, we extract from the global air-travel network the probability of detection $p_{l,l'}$ for each travel $l \to l'$. Each travel $l \to l'$ is associated with a set of potential airport *paths* of the form $\mathcal{P} = \nu_1 \to \nu_2 \to \cdots \to \nu_k$. If the path $\mathcal{P}$ contains an international flight $\nu \to \nu'$, where $\nu' \in \mathcal{S}$, then detectable individuals taking this path will be detected with probability p_{det}. If a path $\mathcal{P}$ contains $\mathcal{N}(\mathcal{P})$ such international flight to any sentinel, the probability of detection is $1 - (1 - p_{\mathrm{det}})^{\mathcal{N}(\mathcal{P})}$. We therefore calculate

$$p_{l,l'} = \sum_{\mathcal{P}} P(\mathcal{P}|l \to l') \left[1 - (1 - p_{\mathrm{det}})^{\mathcal{N}(\mathcal{P})} \right], \tag{4}$$

where $P(\mathcal{P}|l \to l')$ is the probability an individual will take the path $\mathcal{P}$, given the travel $l \to l'$.

1.2 Probability generating function framework

To characterize the early phase of an epidemic, we can map the transmission tree to a branching process, from which an efficient *probability generating function* (PGF) methodology can be leveraged [21–25]. This is a standard

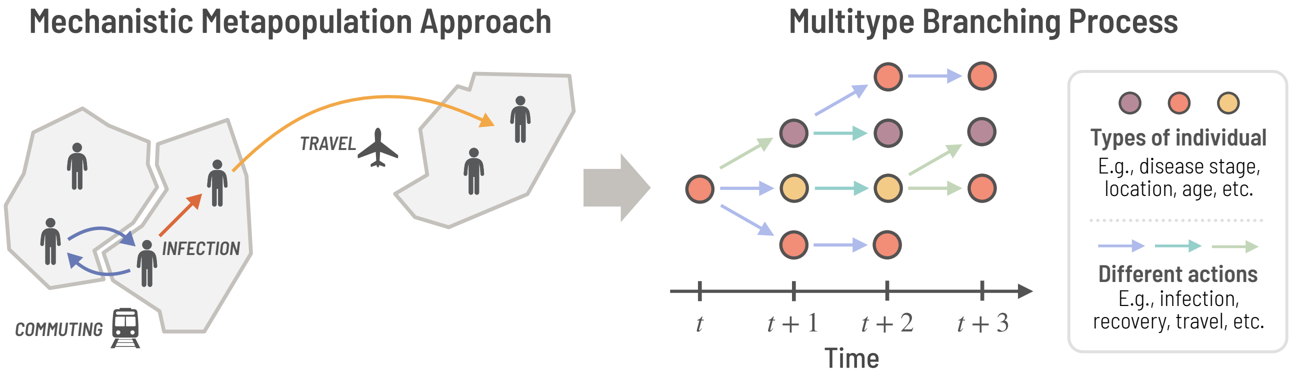

Figure S3: Mapping GLEAM to a multitype branching process. Infections happen within subpopulations—and across adjacent subpopulations when accounting for commuting—and agents can travel between the subpopulations. Individuals are distinguished by their *type*, which encapsulates everything that distinguishes them (like disease stage, age, and location). The early phase of an epidemic naturally takes the form of an event tree encoding all actions happening at each time step, including travel and transmission. This event tree is mathematically described via a multitype branching process.

approach in mathematical epidemiology and has been used, among others, to characterize the time evolution of contagion on heterogeneous networks [26–28], and to quantify the risk of introduction and outbreaks in metapopulation models [29–34]. Here we leverage this methodology by mapping to a multitype branching process the age-structured, stochastic, metapopulation dynamics of GLEAM, the disease progression, and the wastewater surveillance at airports (see Fig. S3).

PGFs are used to encode discrete probability distributions with functions. In the present case, we want to encode the full distribution for the state of the epidemic at all times t—the number of days since the start of the epidemic. The state of the epidemic includes the number of individuals in each stage of the disease, of each age, and in each subpopulation, but also other quantities we want to "measure" (evaluate an associated probability distribution), like the cumulative number of exported cases and the cumulative number of detections on each origin-destination travel ending at a sentinel.

To introduce our formalism, let us define the multi-index $\alpha = (l, a)$ characterizing the location l and the age a of an individual—we refer to α as the *category* of an individual or agent. We define ℓ_α as the number of latent individuals of category α. Since we divide the number of infectious and post-infectious states into two stages (I_1,I_2,P_1, and P_2) we identify them by the numbers $i_{\alpha,1}$, $i_{\alpha,2}$, and $j_{\alpha,1}$,$j_{\alpha,2}$ respectively. To track the number of exported cases, we introduce e_α as the cumulative number of α-agents, either latent or infectious, who were infected previously in another location—they were previously of another category—then traveled, thereby becoming of category α. Finally, to track wastewater detections of detectable individuals (infectious or post-infectious) at sentinel airports, we define $d_{\alpha,\alpha'}$ the cumulative number of detections for international travel $l \to l'$ resulting in a change of category $\alpha \to \alpha'$ for a given age group a. Note that we do not track susceptible or recovered individuals in this framework, since they do not play a significant role in the early phase of an outbreak. The whole state of the system is then described by the tuple of vectors ($\boldsymbol{\ell}$, $\boldsymbol{i}_1$, $\boldsymbol{i}_2$, $\boldsymbol{j}_1$, $\boldsymbol{j}_2$, $\boldsymbol{e}$, $\boldsymbol{d}$), where for instance $\boldsymbol{\ell} = [l_{\alpha_1}, l_{\alpha_2}, \dots]$.

To simplify the notation, we define the *state vector*

$$\boldsymbol{s} = [l_{\alpha_1}, l_{\alpha_2}, \dots, i_{\alpha_1,1}, i_{\alpha_2,2}, \dots, d_{\alpha_1,\alpha_2}, d_{\alpha_1,\alpha_3}, \dots] \equiv [s_1, s_2, \dots] \,, \tag{5}$$

as the concatenation of previous vectors, encoding all information about the system. Each component s_σ of the vector identifies a number we keep track of (e.g., number of infectious of a specific category α) and is associated with nodes

of a specific color in Fig. S3; we refer to σ as the *type* of each node in the multitype branching process representation of the epidemic and mobility dynamics.

Since the state vector is a random variable $\boldsymbol{s}_t$ at each time t, we write the probability of observing a particular state at time t as $P(\boldsymbol{s}_t = \boldsymbol{s}) \equiv P(\boldsymbol{s}, t)$. Finally, we encode this distribution with a PGF (in the compact format) as

$$\Psi^t(\boldsymbol{x}) \equiv \sum_{\boldsymbol{s}} P(\boldsymbol{s}, t) \prod_{\sigma} x_{\sigma}^{s_\sigma} \ , \tag{6}$$

where the sum runs over all potential values of the vector $\boldsymbol{s}$ and each x_σ is a dummy variable used to track each quantity s_σ.

If we unravel the vector $\boldsymbol{s}$, the PGF is written as

$$\Psi^t(\boldsymbol{z}, \boldsymbol{y}_1, \boldsymbol{y}_2, \boldsymbol{w}_1, \boldsymbol{w}_2, \boldsymbol{v}, \boldsymbol{u}) = \sum_{\boldsymbol{\ell}} \sum_{\boldsymbol{i}_1} \sum_{\boldsymbol{i}_2} \sum_{\boldsymbol{j}_1} \sum_{\boldsymbol{j}_2} \sum_{\boldsymbol{e}} \sum_{\boldsymbol{d}} P(\boldsymbol{\ell}, \boldsymbol{i}_1, \boldsymbol{i}_2, \boldsymbol{j}_1, \boldsymbol{j}_2, \boldsymbol{e}, \boldsymbol{d}, t) \prod_{\alpha, \alpha'} z_\alpha^{\ell_\alpha} y_{\alpha,1}^{i_{\alpha,1}} y_{\alpha,2}^{i_{\alpha,2}} w_{\alpha,1}^{j_{\alpha,1}} w_{\alpha,2}^{j_{\alpha,2}} v_\alpha^{e_\alpha} u_{\alpha,\alpha'}^{d_{\alpha,\alpha'}} \ , \tag{7}$$

where the vector of variables $(\boldsymbol{z}, \boldsymbol{y}_1, \boldsymbol{y}_2, \boldsymbol{w}_1, \boldsymbol{w}_2, \boldsymbol{v}, \boldsymbol{u})$ tracks the quantities $(\boldsymbol{\ell}, \boldsymbol{i}_1, \boldsymbol{i}_2, \boldsymbol{j}_1, \boldsymbol{j}_2, \boldsymbol{e}, \boldsymbol{d})$. For obvious reasons, we will favor the compact representation as much as possible, and use the unraveled vectors only when necessary to specify operations on a subset of the variables.

For a general multitype branching process, the solution is obtained by recursion [26]

$$\Psi^{t+1}(\boldsymbol{x}) = \Psi^t \left[\boldsymbol{F}(\boldsymbol{x})\right] \ , \tag{8}$$

where $F(\boldsymbol{x}) = [F_1(\boldsymbol{x}), F_2(\boldsymbol{x}), \dots]$ is a vector of PGFs, and each PGF $F_\sigma(\boldsymbol{x})$ characterizes the *offspring* distribution at the next time step for each node of type σ. For instance, assuming σ identifies infectious α-agents in the first stage (I_1), each individual could lead to a certain number of new latent individuals (through transmission), an α-agent in the second stage (I_2) through disease progression, detection at airports through travel, and so forth. Each possible combination of offspring is encoded in a multivariate PGF, $F_\sigma(\boldsymbol{x})$, similar to Eq. (6).

We now decompose the vector of offspring PGFs $\boldsymbol{F}(\boldsymbol{x})$ by describing separately the reaction phase—modeling disease transmission and progression—, and the mobility phase of GLEAM in terms of vectors of PGFs $\boldsymbol{R}(\boldsymbol{x})$ and $\boldsymbol{M}(\boldsymbol{x})$ respectively. From a similar argument justifying Eq. (8), the vector of offspring PGFs is the composition of the PGFs for each phase [26], i.e., $\boldsymbol{F}(\boldsymbol{x}) = \boldsymbol{M}(\boldsymbol{R}(\boldsymbol{x}))$. The order of the composition indicates that air travel is happening *before* the reaction phase, in line with the convention in GLEAM.

Reaction phase: disease transmission and progression

While the PGF framework Eq. (8) is defined at a daily resolution, we model the reaction phase at a finer temporal resolution by dividing each day into $m = 12$ time periods of duration $\Delta t = 1/m$, as in the GLEAM simulation procedure. This provides a more realistic description of the intraday dynamics, especially for rapidly evolving epidemics, but one could work at any temporal resolution without significantly affecting the results. Consequently, the vector of PGFs $\boldsymbol{R}(\boldsymbol{x})$ for the reaction phase itself corresponds to the m-th composition of the vector of PGFs $\boldsymbol{r}(\boldsymbol{x})$, i.e.,

$$\boldsymbol{R}(\boldsymbol{x}) = \underbrace{\boldsymbol{r}(\boldsymbol{r}(\dots \boldsymbol{r}(}_{m \text{ times}}\boldsymbol{x})\dots)) \ . \tag{9}$$

Each transition in the disease progression has a rate: latent individuals become infectious at rate η, infectious individuals in the first stage transition to the second stage (and then in the post-infectious stage) at rate μ, post-infectious individuals in the first stage transition to the second stage (and then in the removed stage) at rate φ. Also, infectious α-agents (at any stage) interact and transmit the disease to α'-susceptible individuals at rate $\beta_{\alpha,\alpha'}$, which is constructed from the age-structured contact matrix $\mathbf{C}$ and take into account commuting.

Table S2: Offspring PGFs associated with a single step of the reaction phase for each element of the state vector. The parameters η, μ, ϕ correspond to spontaneous transition rates between the disease stages (see Fig. S2). The parameter $\beta_{\alpha,\alpha'}$ is the rate at which infectious α-agents interact and transmit the disease to α'-susceptible individuals.

Element identified by the type σ	Reaction phase PGF $r_\sigma(\boldsymbol{x})$
Latent individuals of category α	$y_{\alpha,1}\eta\Delta t + z_\alpha(1-\eta\Delta t)$
Infectious individuals of category α in the first stage	$\exp\left[\sum_{\alpha'} \beta_{\alpha,\alpha'}(z_{\alpha'}-1)\Delta t\right]\left[y_{\alpha,2}\mu\Delta t + y_{\alpha,1}(1-\mu\Delta t)\right]$
Infectious individuals of category α in the second stage	$\exp\left[\sum_{\alpha'} \beta_{\alpha,\alpha'}(z_{\alpha'}-1)\Delta t\right]\left[w_{\alpha,1}\mu\Delta t + y_{\alpha,2}(1-\mu\Delta t)\right]$
Post-infectious individuals of category α in the first stage	$w_{\alpha,2}\varphi\Delta t + w_{\alpha,1}(1-\varphi\Delta t)$
Post-infectious individuals of category α in the second stage	$\varphi\Delta t + w_{\alpha,2}(1-\varphi\Delta t)$
Exported individuals of category α	v_α
Wastewater detections related to a change of category $\alpha \to \alpha'$	$u_{\alpha,\alpha'}$

Table S3: Offspring PGFs associated with the mobility phase for each element of the state vector. The parameter $m_{\alpha,\alpha'}$ is the probability per day for α-agents to move and become of category α'; the parameter $p_{\alpha,\alpha'}$ is the probability of detection for detectable individuals if they move according to $\alpha \to \alpha'$.

Element identified by the type σ	Mobility phase PGF $M_\sigma(\boldsymbol{x})$
Latent individuals of category α	$\sum_{\alpha'\neq\alpha} m_{\alpha,\alpha'} z_{\alpha'} v_{\alpha'} + m_{\alpha,\alpha} z_\alpha$
Infectious individuals of category α in the first stage	$\sum_{\alpha'\neq\alpha} m_{\alpha,\alpha'} y_{\alpha',1} v_{\alpha'}(1-p_{\alpha,\alpha'}+p_{\alpha,\alpha'}u_{\alpha,\alpha'}) + m_{\alpha,\alpha} y_{\alpha,1}$
Infectious individuals of category α in the second stage	$\sum_{\alpha'\neq\alpha} m_{\alpha,\alpha'} y_{\alpha',2} v_{\alpha'}(1-p_{\alpha,\alpha'}+p_{\alpha,\alpha'}u_{\alpha,\alpha'}) + m_{\alpha,\alpha} y_{\alpha,2}$
Post-infectious individuals of category α in the first stage	$\sum_{\alpha'\neq\alpha} m_{\alpha,\alpha'} w_{\alpha',1}(1-p_{\alpha,\alpha'}+p_{\alpha,\alpha'}u_{\alpha,\alpha'}) + m_{\alpha,\alpha} w_{\alpha,1}$
Post-infectious individuals of category α in the second stage	$\sum_{\alpha'\neq\alpha} m_{\alpha,\alpha'} w_{\alpha',2}(1-p_{\alpha,\alpha'}+p_{\alpha,\alpha'}u_{\alpha,\alpha'}) + m_{\alpha,\alpha} w_{\alpha,2}$
Exported individuals of category α	v_α
Wastewater detections related to a change of category $\alpha \to \alpha'$	$u_{\alpha,\alpha'}$

Considering all these possible transitions, we report in Table S2 the offspring PGF associated to a single step of the reaction phase, $r_\sigma(\boldsymbol{x})$, for each type of node. Disease progression transitions are represented by multinomial PGFs, while disease transmission (for infectious individuals only) is represented by a multivariate Poisson PGF. The multivariate Poisson PGF corresponds to an infinite-size subpopulation approximation for binomial draws used in GLEAM simulations for the infection process. Note that the offspring PGFs for elements of type σ identifying a cumulative number of exported individuals or a cumulative number of wastewater detections at airports are simply the identity PGF, i.e., $f(x) = x$. Indeed, these quantities do not generate new infections or transition to other states, they only serve to keep track of a sum [26].

Mobility phase: air-travel and detection at airports

Each day, latent, infectious, and post-infectious α-agents in location l move to a new subpopulation l' of multi-index α' with probability $m_{\alpha,\alpha'}$ and stay with probability $m_{\alpha,\alpha}$; agents that move and are detectable (infectious or post-infectious) are detected with probability $p_{\alpha,\alpha'} = p_{l,l'}$, calculated in Eq. (4). Similarly to the reaction phase, we report in Table S3 the offspring PGF associated with the mobility phase, $M_\sigma(\boldsymbol{x})$, for each type of node. Except for the types identifying exported individuals and wastewater detections, $M_\sigma(\boldsymbol{x})$ is a multinomial PGF.

Initial conditions

The solution of Eq. (8) takes the form

$$\Psi^t(\boldsymbol{x}) = \Psi^0(\underbrace{\boldsymbol{F}(\boldsymbol{F}(\cdots\boldsymbol{F}(}_{t \text{ times}}\boldsymbol{x})\cdots))) \,. \tag{10}$$

The PGF $\Psi^0(\boldsymbol{x})$ specifies the initial condition. In all cases in this study, we specify a number ℓ and i_1 of initial latent and infectious (I_1) individuals respectively. Their age is randomly selected according to the age distribution of the origin subpopulation l, which we represent by the conditional category distribution $P(\alpha|l)$. The PGF for the initial conditions then takes the form

$$\Psi^0(\boldsymbol{x}) = \left(\sum_\alpha P(\alpha|l) z_\alpha\right)^\ell \left(\sum_\alpha P(\alpha|l) y_{\alpha,1}\right)^{i_1} , \tag{11}$$

It is worth stressing that since Ψ^0 is applied *last* when evaluating Eq. (10), it is computationally inexpensive to test various initial conditions, which we use in this study to vary the origin of the outbreak.

Evaluation of probability distributions and cumulants

For a large number of multi-indices σ, evaluating the full joint distribution $P(\boldsymbol{s}, t)$ is computationally prohibitive. However, it is not our goal: In general, we want to evaluate a marginal or a joint distribution of some *observables*, like d_t, the total number of detections at any sentinels by time t, which can be estimated from the PGF $\Psi^t(\boldsymbol{x})$. For instance, the PGF for the distribution $P(d_t = d)$, is

$$\zeta^t(x) \equiv \sum_d P(d_t = d) x^d = \Psi^t\left[\boldsymbol{A}(x)\right] , \tag{12}$$

where $\boldsymbol{A}(x)$ is a vector where $A_\sigma(x) = x$ if σ identifies any of the cumulative detection at a sentinel for a specific pair of category, $d_{\alpha,\alpha'}$, and $A_\sigma(x) = 1$ otherwise. This allows us to implicitly sum over all possible combinations of $d_{\alpha,\alpha'}$ resulting in a total number of detection d_t. PGFs $\zeta^t(x, y)$ for joint distributions of observables are constructed in similar fashion.

For any observable n_t, we can extract the distribution $P(n_t)$ from its PGF $\zeta^t(x) = \sum_n P(n_t = n) x^n$ using the following identity

$$\begin{aligned} P(n_t = n) &= \frac{1}{n!} \frac{\mathrm{d}^n}{\mathrm{d}x^n} \zeta^t(x) \bigg|_{x=0} , \\ &= \frac{1}{2\pi\rho^n} \int_0^{2\pi} \zeta^t\left(\rho e^{i\omega}\right) e^{-i\omega n} \mathrm{d}\omega , \end{aligned} \tag{13}$$

where $0 < \rho < 1$ is a free control parameter. In practice, we use the following discrete Fourier transform approximation (efficiently calculated using Fast Fourier Transform algorithms)

$$P(n_t = n) \approx \frac{1}{k\rho^n} \sum_{j=0}^{k-1} \zeta^t\left(e^{2\pi i j/k}\right) e^{-2\pi i j n/k} . \tag{14}$$

Note that it is possible to bound and control the error committed by choosing k and a suitable ρ value [35], making this approximation exact for all practical purposes. An analogous multidimensional discrete Fourier transform numerical solution is used for distributions of joint observables.

Finally, in some cases, it is more convenient and computationally efficient to extract a few cumulants—mean, variance, etc.—instead of the full distribution. Fortunately, the *cumulant generating function* (CGF) $K^t(x)$ for an observable is directly related to its PGF $\zeta^t(x)$ by the relation $K^t(x) = \ln \zeta^t(e^x)$ [36]. Cumulants are extracted from the CGF using a discrete Fourier transform procedure similar to Eq. (14), which is also directly generalizable to higher dimensions (joint cumulants).

Minimal example: SIR dynamics in two subpopulations

To illustrate the full PGF methodology from end to end, let us consider a simpler, minimal, example of two subpopulations and a single age group. Therefore, we only have two categories $\alpha \in \{1, 2\}$ associated with each

subpopulation. We also consider a simpler compartmental model: individuals are either susceptible, infectious, or recovered; since in our framework we do not track susceptible and recovered individuals, we only need to track i_1 and i_2, the number of infectious individuals in each subpopulation. The probability distribution for the state of the epidemic at time t, $P(i_1, i_2, t)$, is encoded with the PGF

$$\Psi^t(\boldsymbol{x}) = \Psi^t(y_1, y_2) = \sum_{i_1=0}^{\infty}\sum_{i_2=0}^{\infty} P(i_1, i_2, t) y_1^{i_1} y_2^{i_2} \ , \tag{15}$$

where y_1 and y_2 are placeholder variables tracking i_1 and i_2 respectively.

We assume that the epidemic starts at time $t = 0$ with 1 infectious individuals in subpopulation 1. From Eq. 11, this implies $\Psi^0(y_1, y_2) = y_1$. Let us now consider the evolution of the system for a single time step. To simplify the expressions, we use $m = 1$ ($\Delta t = 1$) for the temporal scale of the reaction phase. The offspring PGFs associated with the mobility phase are written as follows

$$\boldsymbol{M}(y_1, y_2) = \begin{bmatrix} M_1(y_1, y_2) \\ M_2(y_1, y_2) \end{bmatrix} = \begin{bmatrix} m_{1,1}y_1 + m_{1,2}y_2 \\ m_{2,1}y_2 + m_{2,2}y_2 \end{bmatrix} = \begin{bmatrix} m_{1,1} & m_{1,2} \\ m_{2,1} & m_{2,2} \end{bmatrix} \begin{bmatrix} y_1 \\ y_2 \end{bmatrix} \ . \tag{16}$$

We interpret $M_1(y_1, y_2) = m_{1,1}y_1 + m_{1,2}y_2$ as follows: an infectious individual in subpopulation 1 has a probability $m_{1,1}$ to stay in subpopulation 1 and a probability $m_{1,2} = 1 - m_{1,1}$ to move to subpopulation 2. A similar reasoning holds for $M_2(y_1, y_2)$.

The offspring PGFs associated with the reaction phase are written as follows

$$\boldsymbol{R}(y_1, y_2) = \begin{bmatrix} R_1(y_1, y_2) \\ R_2(y_1, y_2) \end{bmatrix} = \begin{bmatrix} \exp\{\beta_{1,1}(y_1 - 1)\} \exp\{\beta_{1,2}(y_2 - 1)\}\{\mu + (1-\mu)y_1\} \\ \exp\{\beta_{2,1}(y_1 - 1)\} \exp\{\beta_{2,2}(y_2 - 1)\}\{\mu + (1-\mu)y_2\} \end{bmatrix} \ . \tag{17}$$

We interpret $R_1(y_1, y_2)$ as follows: 1) An infectious individual in subpopulation 1 will generate a number of secondary infections in subpopulation 1, distributed according to a Poisson distribution with mean $\beta_{1,1}$, to which corresponds the PGF $\exp\{\beta_{1,1}(y_1 - 1)\}$. 2) If we take into account commuting, i.e., $\beta_{1,2} > 0$, an infectious individual in subpopulation 1 will also generate secondary infections in subpopulation 2, with PGF $\exp\{\beta_{1,2}(y_2 - 1)\}$. 3) An infectious individual in subpopulation 1 will either recover with probability μ or remain infectious with probability $1 - \mu$, resulting in the PGF $\mu + (1 - \mu)y_1$. Since these 3 kinds of events are *independent*, the PGF for the sum is the product of the individual PGFs [25], resulting in the expression for $R_1(y_1, y_2)$. A similar reasoning holds for $R_2(y_1, y_2)$.

Combining the mobility and reaction phases, the offspring PGFs $\boldsymbol{F}(\boldsymbol{x}) = \boldsymbol{M}(\boldsymbol{R}(\boldsymbol{x}))$ for infectious individuals in both subpopulations are

$$\boldsymbol{F}(y_1, y_2) = \begin{bmatrix} F_1(y_1, y_2) \\ F_2(y_1, y_2) \end{bmatrix} = \begin{bmatrix} M_1(R_1(y_1, y_2), R_2(y_1, y_2)) \\ M_2(R_1(y_1, y_2), R_2(y_1, y_2)) \end{bmatrix} = \begin{bmatrix} m_{1,1} & m_{1,2} \\ m_{2,1} & m_{2,2} \end{bmatrix} \begin{bmatrix} R_1(y_1, y_2) \\ R_2(y_1, y_2) \end{bmatrix} \ . \tag{18}$$

Using the recursion in Eq. (8) and the offspring PGFs above, we are able to calculate the PGF for the state of the epidemic at time $t = 1$

$$\Psi^1(y_1, y_2) = \Psi^0(F_1(y_1, y_2), F_2(y_1, y_2)) = F_1(y_1, y_2) = m_{1,1}R_1(y_1, y_2) + m_{1,2}R_2(y_1, y_2) \ . \tag{19}$$

We interpret Eq. (19) as follows: with probability $m_{1,1}$, the index case will stay in the first subpopulation, and generate secondary infections as prescribed by $R_1(y_1, y_2)$, or travel to subpopulation 2 with probability $m_{1,2}$ and generate secondary infections as prescribed by $R_2(y_1, y_2)$.

From $\Psi^1(y_1, y_2)$, we can estimate any distributions of *observables* for the state of the epidemic at time $t = 1$. For instance, if we compose Ψ^1 with $\boldsymbol{A}(x) = [A_1(x), A_2(x)]^\top = [x, x]^\top$ to get the PGF

$$\zeta^1(x) = \Psi^1(x, x) = m_{1,1}R_1(x, x) + m_{1,2}R_2(x, x) \ , \tag{20}$$

then $\zeta^1(x)$ encodes the distribution for the total number of infectious in both subpopulations, which can be recovered numerically through Eq. (14).

Altogether, this minimal example with the SIR model in two subpopulations illustrates the full PGF methodology: We start from a PGF describing the initial conditions, $\Psi^0(\boldsymbol{x})$, we define the vector of offspring PGFs $\boldsymbol{F}(\boldsymbol{x})$, using Eq. (8) we get the PGF for the state of the epidemic at a later time $\Psi^t(\boldsymbol{x})$, and finally we use it to get a PGF for the distribution of an observable $\zeta^t(x)$, like the total number of infectious individuals. For the compartmental model in Fig. S2 and the metapopulation network of GLEAM, the dimension of the PGFs and equations is much larger, but the procedure is exactly the same.

2 Time to first detection for a global wastewater surveillance system at airports

An important quantity introduced in the main text is the time to first detection, t_{fd}, with distribution

$$P(t_{\text{fd}} = t) = P(d_{t-1} < 1, d_t \geq 1) \; . \tag{21}$$

We can simplify the joint distribution on the right-hand side. Note that the statements $A \equiv d_{t-1} < 1$ and $B \equiv d_t \geq 1$ are Boolean random variables. We can write the probability $P(A, B) = P(A) - P(A, \neg B)$. Since $P(\neg A, \neg B) = 0$ because it is impossible, then $P(A, \neg B) = P(\neg B)$. Therefore,

$$P(d_{t-1} < 1, d_t \geq 1) \equiv P(A) - P(\neg B) = P(d_{t-1} < 1) - P(d_t < 1) = P(d_{t-1} = 0) - P(d_t = 0) \; , \tag{22}$$

which is straightforward to evaluate using the PGFs. Let us emphasize that the time to first detection is independent of the wastewater methodology being used (pooled sampling or individual aircraft sampling), in the sense that it does not matter if more than one detectable individual contributed to a positive test at a sentinel. It is also worth highlighting the close connection with the extensive line of work on the *arrival time* of disease in metapopulation networks [37–41].

To complement Fig. 1 in the main text, we show in Fig. S4 the mean time to first detection from all potential sources for the four different levels of detection probability (4%, 8%, 16%, and 32%). We additionally validate in Fig. S5 the PGF solutions with GLEAM simulations for the full distribution of t_{fd} for two epidemic origins. In Fig. S5(A), the PGF prediction accurately reproduces the distribution, with T_{fd}, the mean time to first detection, being within less than 1 standard error (SE) of the simulation estimate. In Fig. S5(B), there are approximately 2 days of difference between the two estimates of T_{fd} (i.e., 3% difference), which is due to finite subpopulation effects not taken into account by the PGFs. Note that Kalemie is a subpopulation for which it takes a long time to detect with this surveillance system and therefore accentuates the discrepancy.

In Fig. 2 of the main text, we present results for T_{fd} aggregated over continents; in Fig. S6, we present a similar same analysis, but aggregating over statistical subregions in Africa and Asia. We again observe broad heterogeneity of the mean time to first detection at this geographical scale in all subregions.

An important surveillance network introduced in the main text is the complete WWSN, where all airports act as surveillance sites. In Fig S7, we show T_{fd} from every potential origin in the world for the complete WWSN. We note the similarity with the global map obtained in Figs. 1. The mean time to first detection is still very heterogeneous, with some locations taking a week and others as much as 100 days before a first detection by the WWSN. To better explain the source of this heterogeneity, in Fig. S8, we show the relation between the probability per day of traveling to an international destination for an individual in each location and T_{fd}. There is a clear negative correlation between the two—a higher probability leads to a faster detection—, but other factors are important. For instance, for some subpopulations, international travel is not directly possible—an individual must first travel to another subpopulation within the same country before moving abroad. In other words, one must take into account additional seeding events through importations or commuting to fully characterize the time to first detection.

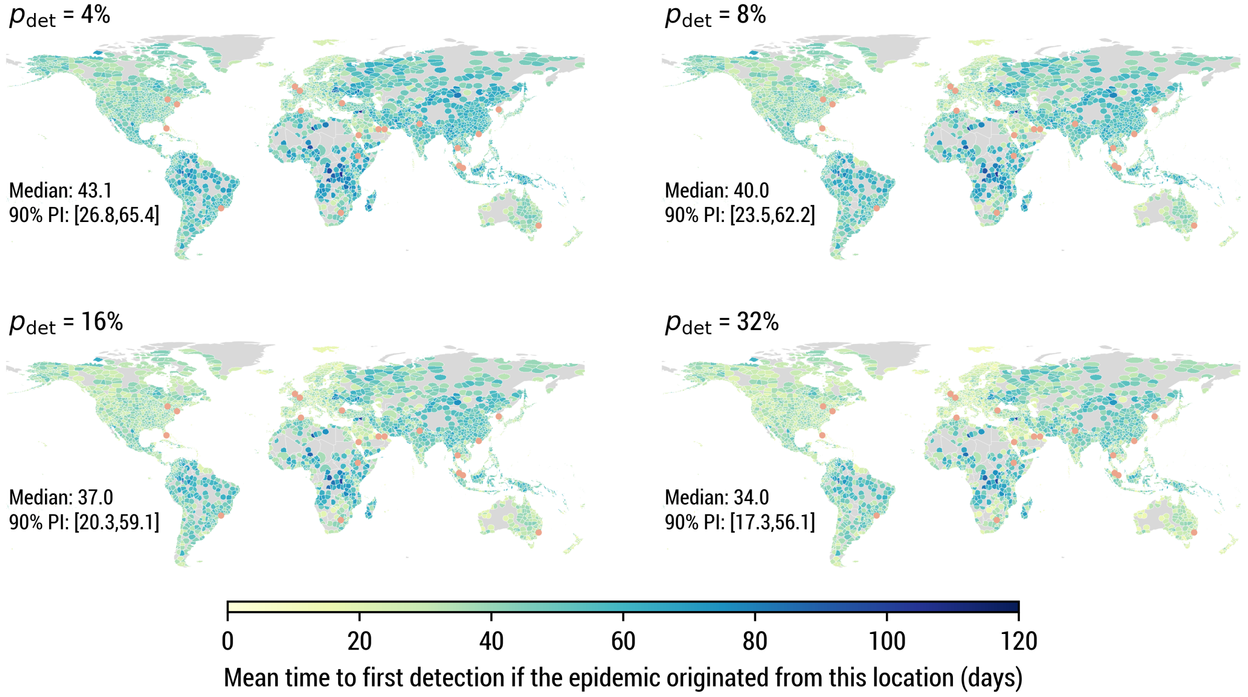

Figure S4: Mean time to first detection using different detection probabilities p_{det} at the sentinels. Otherwise, we use the same WWSN and model parametrization as in Fig. 1. Statistics reported are based on $n = 3244$ subpopulations.

2.1 Variation with the disease natural history

Changing the disease's natural history affects significantly T_{fd}, as can be seen in Fig. 4 in the main text. For the sake of completeness, we also show in Fig. S9 a similar analysis, where instead of varying generation time via the latent period, we do it via the infectious period, keeping the detectable period fixed. We obtain similar results for the mean time to first detection as in Fig. 4.

By changing the generation time or the reproduction number, we ultimately change the growth rate λ of the epidemic—or equivalently the doubling time $T_2 \equiv \ln 2/\lambda$. For the model we consider, the growth rate can be obtained by solving the following implicit nonlinear equation [11]:

$$\mathcal{R}_0 = \lambda T_{\text{inf}} \frac{\left(1 + \frac{\lambda T_{\text{lat}}}{n_{\text{lat}}}\right)^{n_{\text{lat}}}}{\left[1 - \left(1 + \frac{\lambda T_{\text{inf}}}{n_{\text{inf}}}\right)^{-n_{\text{inf}}}\right]} , \tag{23}$$

where n_{lat} and n_{inf} are the numbers of latent and infectious states respectively (here we use $n_{\text{lat}} = 1$ and $n_{\text{inf}} = 2$).

In Fig. 4 of the main text, we show that for all practical purposes, the following relationship holds:

$$\frac{T_{\text{fd}}}{T_2} + \log_2 T_2 \approx \text{const.} \tag{24}$$

Another way to interpret this equation is that changing the doubling time from T_2 to $\hat{T}_2$ ultimately amounts to a linear transformation of the form

$$\hat{T}_{\text{fd}} = aT_{\text{fd}} + b \quad ; \quad a = \left(\frac{\hat{T}_2}{T_2}\right) \quad ; \quad b = \hat{T}_2 \log_2 \left(\frac{T_2}{\hat{T}_2}\right) , \tag{25}$$

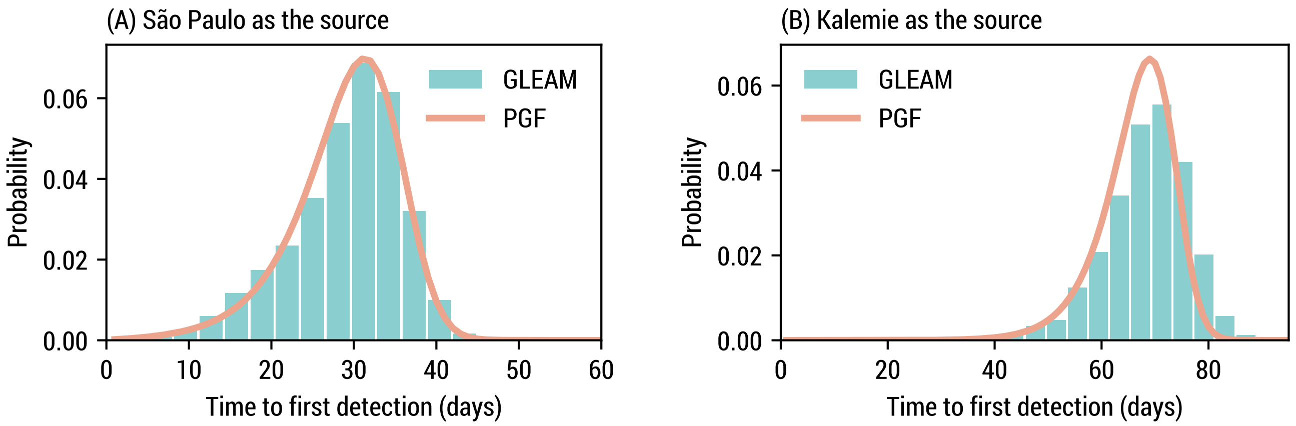

Figure S5: Comparison of the time to first detection. We use the same model, parametrization, and baseline WWSN as in Fig. 1. (A) The epidemic starts in São Paulo. GLEAM estimates are based on 1135 simulation runs. The mean time to first detection is 29.98 (SE, 0.19) days for GLEAM and 29.97 days using the PGFs. (B) The epidemic starts in Kalemie. GLEAM estimates are based on 1590 simulation runs. The mean time to first detection is 68.47 (SE, 0.20) days for GLEAM and 66.46 days using the PGFs.

where $\hat{T}_{\text{fd}}$ is the mean time to first detection with doubling time $\hat{T}_2$. This is illustrated in Fig. S10 for all potential origins, when changing the mean generation time or the reproduction number. The Pearson correlation coefficients are very high (>0.99).

To justify Eq. (24), let us rephrase more formally the argument introduced in the main text. If we neglect the stochastic fluctuations of the epidemic, the number of detectable individuals is approximately $D(t) \simeq D_0 e^{\lambda t}$, t days after the beginning of the outbreak. Let us also assume that each day, a detectable individual has some constant probability ω to travel and be detected by the WWSN. In that case, we can approximate the probability of having a first detection on day t as [37]

$$P(t_{\text{fd}} = t) \approx [1 - (1-\omega)^{D(t)}] \prod_{t'=1}^{t-1} (1-\omega)^{D(t')} \tag{26}$$

$$\approx \xi e^{\lambda t} \exp\left(-\frac{\xi}{\lambda} e^{\lambda t}\right) , \tag{27}$$

where $\xi = \omega D_0$, and the approximations hold when $\xi/\lambda \ll 1$. Since the probability per day of air travel is very small in general (see Fig. S8), this approximation is almost always valid. We recognize a Gumbel distribution with mean

$$T_{\text{fd}} = \frac{1}{\lambda}\left[\ln\left(\frac{\lambda}{\xi}\right) - \gamma\right] , \tag{28}$$

where γ is the Euler–Mascheroni constant. Rearranging the terms and using $\lambda = \ln 2/T_2$, we get

$$\frac{T_{\text{fd}}}{T_2} + \log_2 T_2 = \frac{1}{\ln 2}(\ln(\ln 2) - \ln \xi - \gamma) = \text{const.} , \tag{29}$$

which is identical to Eq. (24).

In Fig. S11, we show that the Gumbel distribution approximates well the distribution for the time to first detection, especially when the initial number of latent and infectious is high in Fig. S11(B). This is due to the fact that the Gumbel approximation neglects the stochastic fluctuations of the epidemic, which are more important when starting with fewer exposed individuals.

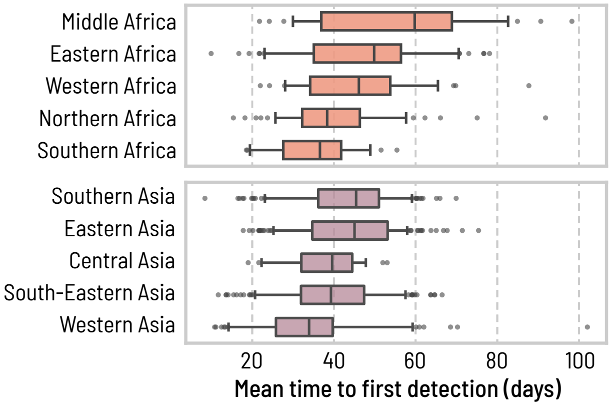

subregion	min	P5	median	mean	P95	max
M. Africa	21.8	28.2	59.8	55.8	84.5	98.3
E. Africa	9.9	23.0	49.9	47.2	70.5	78.1
W. Africa	22.0	28.0	46.1	46.2	67.2	87.7
N. Africa	15.4	24.5	38.4	40.1	58.7	91.8
S. Africa	18.6	19.1	36.6	35.6	50.2	55.4
S. Asia	8.4	23.1	45.4	43.6	59.1	69.9
E. Asia	17.8	24.9	45.0	43.9	58.1	75.4
C. Asia	19.0	22.1	39.6	37.7	48.6	53.0
S.-E. Asia	11.7	20.6	39.3	39.6	57.7	66.5
W. Asia	10.8	14.2	33.9	34.1	59.3	102.1

Figure S6: Heterogeneity of the time to first detection within statistical subregions. We aggregate the mean time to first detection T_{fd} obtained in Fig. 1 over statistical subregions as defined by the United Nations geoscheme in Africa (Middle, $n = 45$; Eastern $n = 121$; Western $n = 52$; Northern $n = 89$; Southern $n = 31$) and Asia (Southern $n = 205$; Eastern $n = 269$; Central $n = 37$; South-Eastern $n = 236$; Western $n = 120$). The center line of the box plot indicates the median, the box covers the interquartile range, the whiskers cover the 90% central prediction interval (P5–P95), and black dots correspond to outliers outside this interval. Numerical values for some of the statistics of the mean time to first detection are reported in the table on the right.

To obtain the Gumbel approximations in Fig. S11, we calculate λ and T_{fd} from our PGF framework, which allows us to fix ξ in Eq. (28). While ξ should not depend on λ, we do find small variations, which also explain the small variations of the approximate invariant quantity in Eq. (24) and in Fig. 4. This is because we treated the detectable individuals $D(t)$ as one homogeneous population with a fixed probability of moving and being detected ω, while in fact it is a heterogeneous group of individuals distributed across a complex metapopulation network.

2.2 Sensitivity analyses

Other aspects of the disease natural history

Aside from the reproduction number and the generation time that directly affect the growth rate, other aspects of the disease's natural history impact the time to first detection, but less significantly so. Here we vary the length of the post-infectious period—thereby changing the detectable period—and the shape of the secondary-infection distribution. In our framework, we do not directly fix the secondary-infection distribution, but rather the secondary-infection distribution per time step. To tune the variance, we replace the multivariate Poisson PGF term in the reaction phase (see Table S2) by the composition of a multinomial and a negative binomial PGF, namely

$$\exp\left[\sum_{\alpha'} \beta_{\alpha,\alpha'}(z_{\alpha'} - 1)\Delta t\right] \mapsto \left[1 + \frac{\Delta t}{\kappa}\sum_{\alpha'} \beta_{\alpha,\alpha'}(1 - z_{\alpha'})\right]^{-\kappa} . \tag{30}$$

This results in the number of secondary infections per time step being distributed according to a negative binomial [42], a generalization of the Poisson distribution. The composition with the multinomial PGF ensures contacts are made according to the age-structured contact matrix. Increasing the overdispersion parameter κ reduces the variance while reducing κ increases the variance; in the limit $\kappa \to \infty$, we recover the multivariate Poisson PGF. It is important to distinguish the overdispersion parameter κ given here, associated to the secondary-infection distribution per time step of duration Δt, versus what one would expect using a negative binomial for the secondary-infection distribution over the whole infectious period.

Another way to communicate the overdispersion in epidemiology is to assess what portion of infections are caused

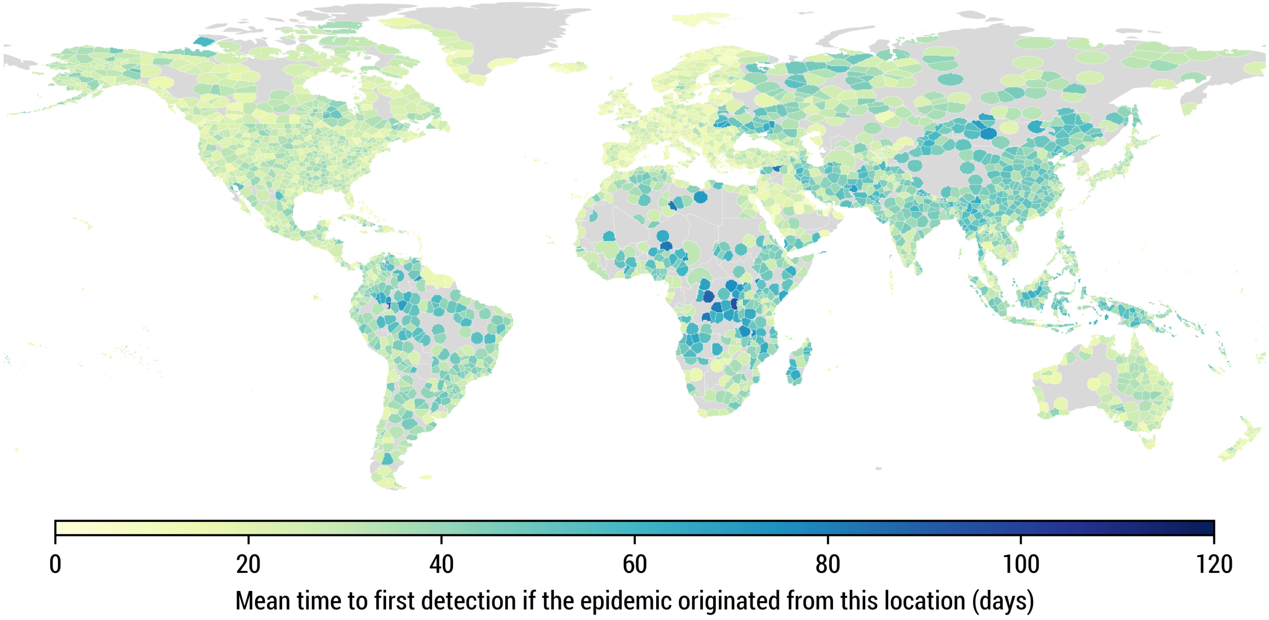

Figure S7: Mean time to first detection with the complete global surveillance network, with a sentinel at every airport. We use the same model parametrization as in Fig. 1.

Table S4: Statistics of the mean time to first detection for various post-infectious periods and overdispersion parameters for the secondary-infection distribution (per time step). The rest of the model parameters and the WWSN are the same as in Fig. 1. We report the median and the 5th and 95th percentile in parentheses ($n = 3244$).

Overdispersion κ		0.01	0.03	0.1	∞
Infections caused by the top 20%		81.9%	66.2%	56.8%	51.3%
Post-Infec. period	5	39.6 (22.4,62.1)	38.3 (21.4,60.7)	37.9 (21.1,60.1)	37.7 (21.0,59.9)
	10	38.9 (21.7,61.4)	37.7 (20.8,60.0)	37.2 (20.4,59.4)	37.0 (20.3,59.1)
	20	38.5 (21.3,60.9)	37.2 (20.4,59.5)	36.8 (20.1,58.9)	36.6 (20.0,58.7)

by the top 20% of infectors [42]. We compile our results in Table S4 for various combinations of post-infectious period and overdispersion parameters. Overall, both have a limited impact on the distribution of T_{fd} for all subpopulations. A broader secondary-infection distribution will mainly affect the beginning of an epidemic—once the outbreak is large enough, the stochastic fluctuations are averaged out. Similar results were obtained in Ref. [6]. The small impact of changing the detectable period (through the post-infectious period) is due to the fact that the number of infectious grows exponentially at the beginning of an epidemic. Consequently, unless the growth rate of the disease is very small, detection at airports should be predominantly caused by newly infectious individuals traveling.

Temporal variation of the traveling patterns

Variation of the air-travel patterns also does not lead to large variations of the global statistics of T_{fd} for all locations, as illustrated in Fig. S12. The median of the distribution for T_{fd} varies from 36 days in the summer to 38 days in the Fall, and the 90% prediction intervals remain relatively stable.

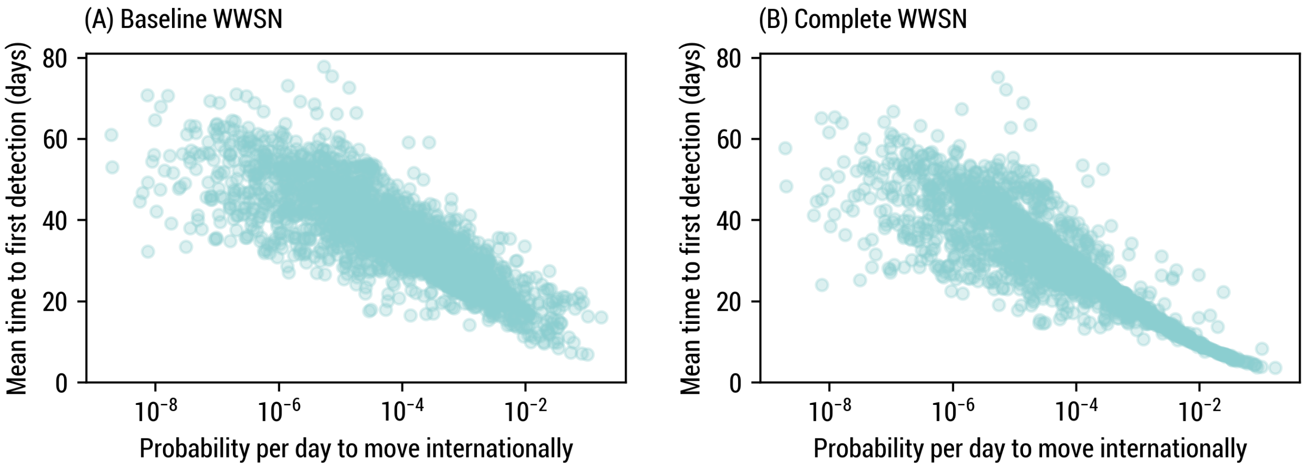

Figure S8: Mean time to first detection for different potential origin, against the probability per day to move internationally from each subpopulation. We only include subpopulations as origin where this probability is larger than zero. We use the same model parametrization as in Fig. 1. (A) We use the same baseline WWSN as in Fig. 1. The Pearson correlation coefficient is -0.810 (90% CI, -0.821 to -0.799; two-sided P value $<10^{-16}$, testing non-correlation) between the mean time to first detection and the logarithm of the probability per day to move internationally ($n = 2509$). (B) We use the complete WWSN, with a sentinel at every airport. The Pearson correlation coefficient is -0.862 (90% CI, -0.870 to -0.853; two-sided P value $<10^{-16}$, testing non-correlation) between the mean time to first detection and the logarithm of the probability per day to move internationally ($n = 2509$).

Heterogeneous detection at sentinel airports

In Fig. S13, we show that the time to first detection is robust to variation of the detection probability across sentinels. Instead of considering a homogeneous detection probability p_{det} at each sentinel, we allow a different probability of detection $p_{\text{det},\nu}$ for each sentinel $\nu \in \mathcal{S}$ (conventionally, we define $p_{\text{det},\nu} = 0$ if $\nu \notin \mathcal{S}$). We model this using a beta distribution with mean $\langle p_{\text{det}} \rangle = 16\%$ and varying standard deviation $\sigma_{p_{\text{det}}}$ in Figs. S13(A) to (C). To account for variable detection probability at each sentinel in our model, we generalize Eq. (4) to

$$p_{l,l'} = \sum_{\mathcal{P}} P(\mathcal{P}|l \to l') \left[1 - \prod_{\nu \in \mathcal{P}} (1 - p_{\text{det},\nu})\right] , \tag{31}$$

for the detection probability of a detectable individual traveling from l to l', where $\mathcal{P} = \nu_1 \to \nu_2 \to \cdots \to \nu_k$ is a potential sequence of airports connecting the two locations.

Even in the case of very heterogeneous detection probabilities ($\sigma_{p_{\text{det}}} = 10\%$), the mean time to first detection from all sources is highly correlated with the homogeneous case, as seen in Fig. S13(F). The geographical variation of the mean time to first detection in Fig. S13(G) is also very similar to the homogeneous case in Fig. 1.

2.3 Optimization of the time to first detection

As illustrated in the main text, ranking airports based on their international inbound passenger volume is a simple, yet effective heuristic to minimize the time to first detection. To further support this point, we present in Fig. S14 an analysis of the mean time to first detection, considering a modified baseline WWSN where instead of systematically selecting the busiest international hubs, we select a combination of busiest and smaller airports in each World Health Organization region. While the detection from a few potential sources is enhanced, overall, the mean time to first detection is longer when discarding large hubs. This reinforces the crucial role of large international hubs in the dissemination of infectious diseases.

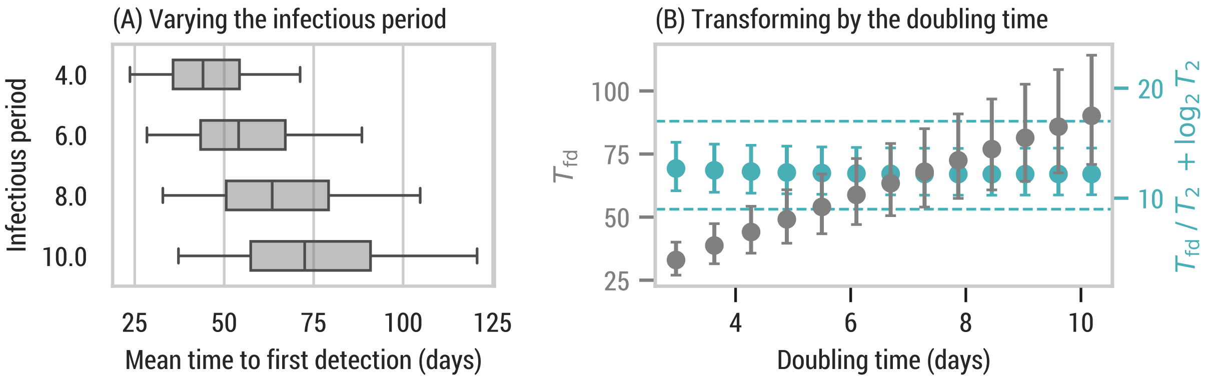

Figure S9: Impact on the mean time to first detection of varying the generation time via the infectious period instead of the latent period. We keep the detectable period fixed to 15 days by also changing the post-infectious detectable period as $T_{\mathrm{post}} = T_{\mathrm{det}} - T_{\mathrm{inf}}$. Otherwise, we use the same WWSN and parametrization as in Fig. 1. All prediction intervals are obtained from $n = 3244$ subpopulations. (A) The center line of the box plot indicates the median, the box covers the interquartile range and the whiskers cover the 90% central prediction interval; the outliers outside the interval are not shown. (B) Circles indicate the median and the error bars cover the interquartile range. The dashed lines are there to guide the eyes. We vary the infectious period between 2 and 14 days, resulting in doubling times between 3 and 10.2 days.

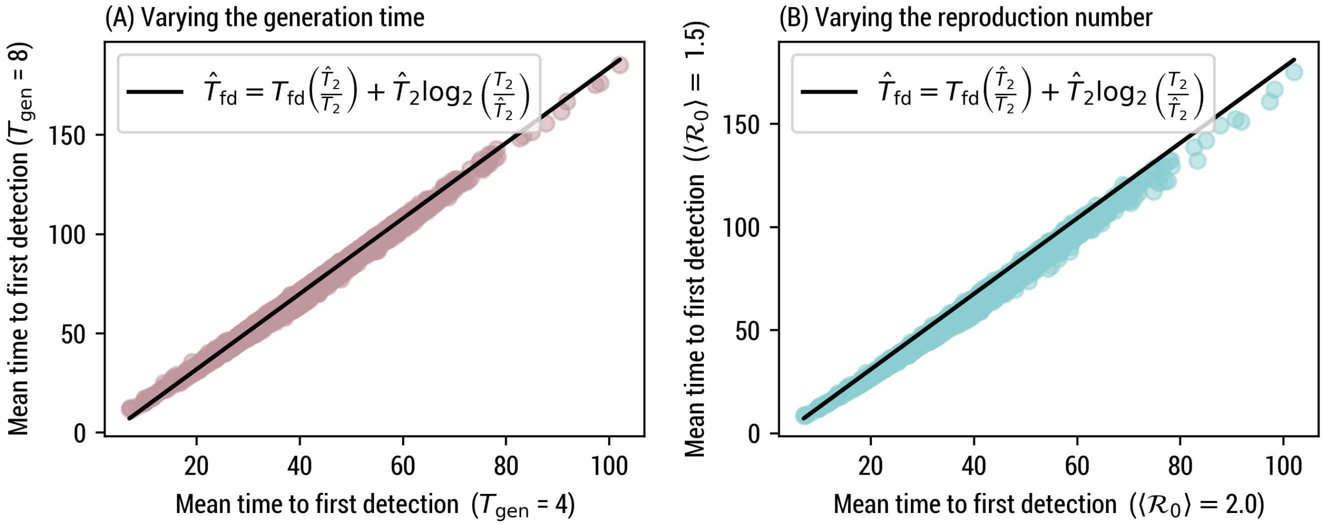

Figure S10: Linear transformation of the mean time to first detection for varying natural disease history. We use the same WWSN as in Fig. 1. The detection probability is 16% and the detectable period is 12.7 days. (A) The mean reproduction number is fixed $\langle \mathcal{R}_0 \rangle = 2$, and the mean generation time varies. The Pearson correlation coefficient is 0.997 with a two-sided P value $<10^{-16}$ ($n = 3244$), testing non-correlation. (B) The mean generation time is fixed $T_{\mathrm{gen}} = 4$, and the reproduction number varies. The Pearson correlation coefficient is 0.996 with a two-sided P value $<10^{-16}$ ($n = 3244$), testing non-correlation.

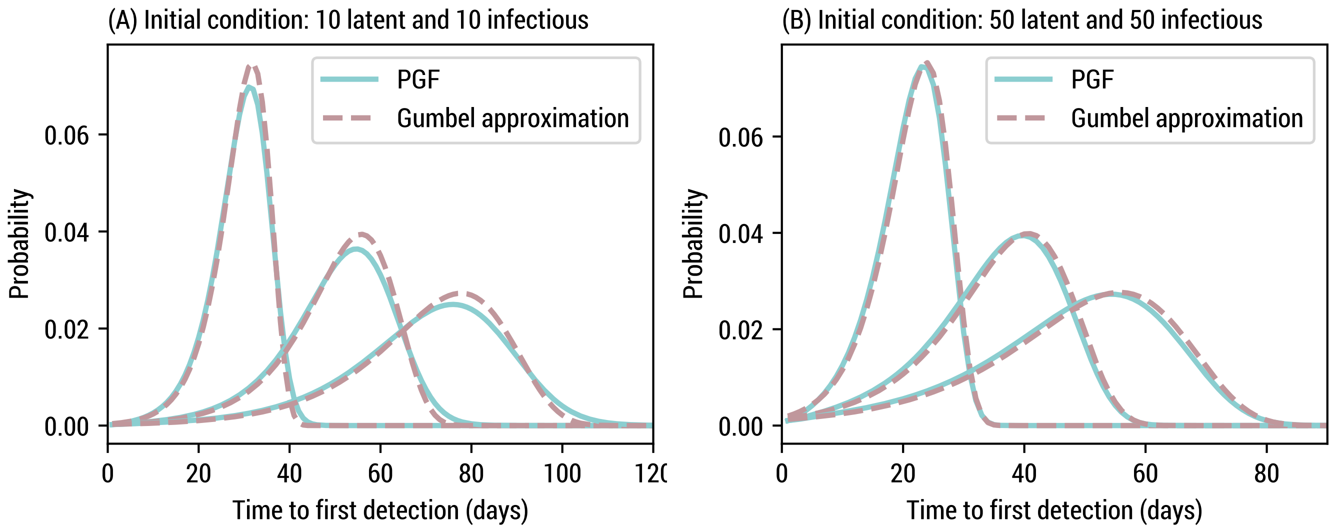

Figure S11: Approximation of the time to first detection using a Gumbel distribution. We use the same WWSN as in Fig. 1 and, unless specified, the same parametrization. We focus on an epidemic originating in São Paulo. We show the results for mean generation times of 4, 8, and 12 days. We consider two initial conditions: (A) 10 latent and 10 infectious individuals and (B) 50 latent and 50 infectious individuals.

In the Methods section of the main text, we introduce a more sophisticated greedy optimization scheme to minimize the mean time to first detection, averaged over all subpopulations as a potential source

$$\Phi(\mathcal{S}) = \sum_{l} P(l) T_{\mathrm{fd}}(\mathcal{S}, l) \,, \tag{32}$$

with probability $P(l)$ of being the source. In the second step of the optimization procedure, it requires the evaluation of $\Phi(\mathcal{S} \cup \{\nu\})$ for each airport $\nu \notin \mathcal{S}$ to assess the reduction in time to first detection associated with adding ν as a sentinel. However, evaluating $\Phi(\mathcal{S} \cup \{\nu\})$ for each new potential sentinel is computationally expensive.

Instead, we use an approximation for each $T_{\mathrm{fd}}(\mathcal{S}, l)$ hinging on two assumptions. First, we assume the full distribution of the time to first detection from each source subpopulation l is well-described by a Gumbel distribution—which is validated in Fig. S11. Second, we assume that the detection at all sentinels $\nu \in \mathcal{S}$ are independent processes. From these assumptions, we can leverage the following identity [37]

$$\exp\left(-\lambda T_{\mathrm{fd}}(\mathcal{S}, l)\right) = \sum_{\nu \in \mathcal{S}} \exp\left(-\lambda T_{\mathrm{fd}}(\{\nu\}, l)\right) \,. \tag{33}$$

This allows us to efficiently estimate $T_{\mathrm{fd}}(\mathcal{S}, l)$ from the individual $T_{\mathrm{fd}}(\{\nu\}, l)$ for all $\nu \in \mathcal{S}$. Figure S15 validates the accuracy of the approximation.

Submodularity proof

In the context of the approximation provided by Eq. (33), we want to show that $-\Phi(\mathcal{S})$ is a monotone submodular set function. Since $-\Phi(\mathcal{S})$ is a positive linear combination of $-T_{\mathrm{fd}}(\mathcal{S}, l)$ for each potential source l, it suffices to show that $-T_{\mathrm{fd}}(\mathcal{S}, l)$ is a monotone submodular set function for all l [44].

We define the set of all potential sentinels as Ω. Let us also simplify the notation by writing $\tau(B) \equiv T_{\mathrm{fd}}(B, l)$ and $\tau(b) \equiv T_{\mathrm{fd}}(\{b\}, l)$ for the mean time to first detection for every set of sentinels $B \subseteq \Omega$ and every sentinel b respectively[1]. Note that $\tau : 2^{\Omega} \to \mathbb{R}^{+}$ is a set function, where 2^{Ω} is the set of all subsets of Ω

[1]The ensuing results are general for all epidemic source l and therefore we drop the index to simplify the notation.

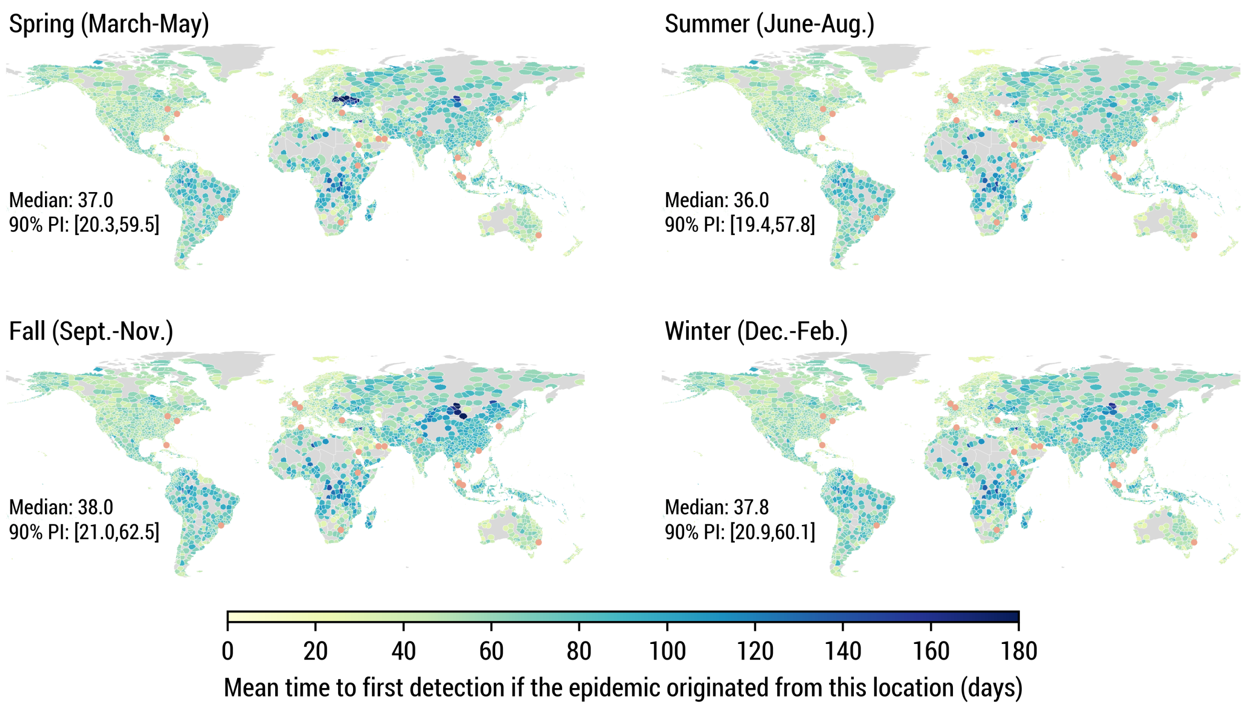

Figure S12: Mean time to first detection using different air-travel mobility networks associated with different seasons. We use the same WWSN and model parametrization as in Fig. 1. Statistics reported are based on $n = 3244$ subpopulations.

Lemma 1. *τ and $-\tau$ are monotone functions, i.e., $\tau(B) \leq \tau(A) \iff -\tau(B) \geq -\tau(A)$ for $A \subseteq B$.*

Proof. Let us define $C \equiv A \cap B$. From Eq. (33), we have

$$e^{-\lambda\tau(B)} = e^{-\lambda\tau(A)} + e^{-\lambda\tau(C)} \geq e^{-\lambda\tau(A)} , \tag{34}$$

where $\lambda > 0$. Taking the logarithm on both sides and dividing by λ, this implies $-\tau(B) \geq -\tau(A)$. □

Definition 1. *Let Ω be a finite set and $f : 2^\Omega \to \mathbb{R}$ a set function. The function f is submodular if for two subsets $A, B \subseteq \Omega$ with $A \subseteq B$ and $e \in \Omega \setminus B$, we have $f(A \cup \{e\}) - f(A) \geq f(B \cup \{e\}) - f(B)$ [44].*

Theorem 1. *The function $-\tau$ is monotone and submodular.*

Proof. The function $-\tau$ is monotone from lemma 1. Let us consider two sets A and B such that $A \subseteq B \subseteq \Omega$, $C \equiv B \setminus A$, and an element $e \in \Omega \setminus B$. Using Eq. (33), we have

$$\begin{aligned} e^{-\lambda\tau(B\cup\{e\})} &= e^{-\lambda\tau(B)} + e^{-\lambda\tau(e)} , \\ &= e^{-\lambda\tau(A)} + e^{-\lambda\tau(C)} + e^{-\lambda\tau(e)} , \end{aligned}$$

where $\lambda > 0$. Dividing both sides by $e^{-\lambda\tau(B)}$ and applying again Eq. (33), we find

$$e^{-\lambda[\tau(B\cup\{e\})-\tau(B)]} = \frac{e^{-\lambda\tau(A\cup\{e\})} + e^{-\lambda\tau(C)}}{e^{-\lambda\tau(A)} + e^{-\lambda\tau(C)}} \leq e^{-\lambda[\tau(A\cup\{e\})-\tau(A)]} , \tag{35}$$

where the inequality holds if and only if $\tau(A \cup \{e\}) \leq \tau(A)$, which is true from lemma 1, but also more intuitively because adding sentinels can only reduce the time to first detection. Taking the logarithm on both sides of Eq. (35)

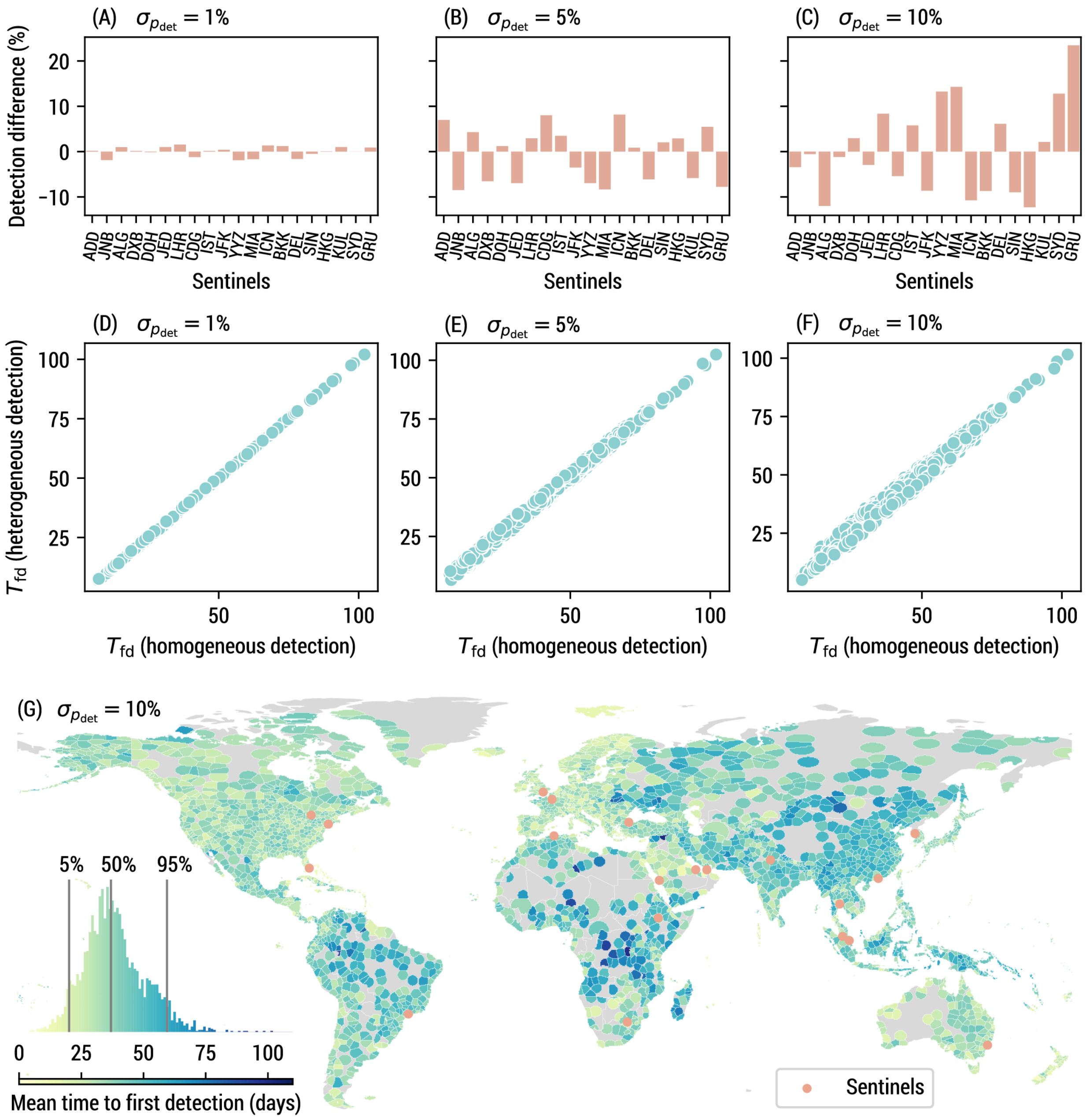

Figure S13: Impact on the mean time to first detection of considering a heterogeneous detection probability at different sentinels. We use the same WWSN and model parametrization as in Fig. 1, but instead of a homogeneous detection probability $p_{\det} = 16\%$, for each sentinel ν we draw a detection probability $p_{\det,\nu}$ from beta distribution with mean $\langle p_{\det} \rangle = 16\%$ and varying standard deviation $\sigma_{p_{\det}}$. (A)-(C) Difference between $p_{\det,\nu}$ and $\langle p_{\det} \rangle = 16\%$ for each sentinel identified by their IATA code [43]. (D)-(F) Comparison of the mean time to first detection from all potential sources, between the homogeneous and heterogeneous cases. The Pearson correlation coefficient is (D) $>$ 0.999, (E) 0.997, (F) 0.993, and the two-sided P value is $<10^{-16}$ in all cases ($n = 3244$), testing non-correlation. (G) Mean time to first detection for the $\sigma_{p_{\det}} = 10\%$ case.

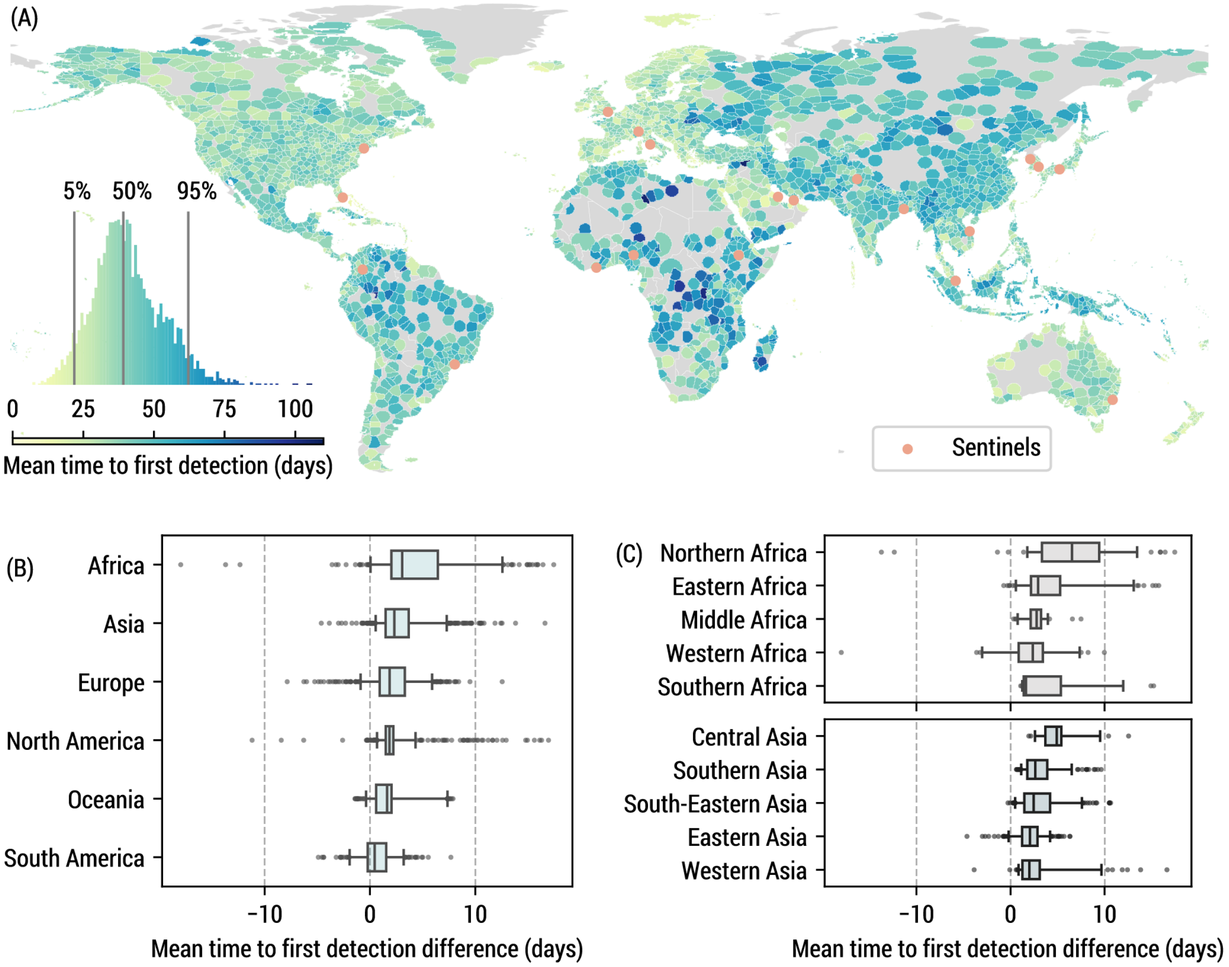

Figure S14: Mean time to first detection with a modified baseline surveillance network, where instead of selecting the three busiest airports in each World Health Organization region, we select the 1st, 10th, and 20th airports ranked by volume of inbound international passengers. We still use the busiest international airports for the two additional sites in South America and Oceania. We use the same model parametrization as in Fig. 1. (A) Mean time to first detection from all potential origins. (B) We aggregate the mean time to first detection over continents (S. America, $n = 297$; Africa $n = 338$; Asia $n = 867$; N. America $n = 854$; Europe $n = 596$; Oceania $n = 292$). (C) We aggregate the mean time to first detection over statistical subregions as defined by the United Nations geoscheme in Africa (Middle, $n = 45$; Eastern $n = 121$; Western $n = 52$; Northern $n = 89$; Southern $n = 31$) and Asia (Southern $n = 205$; Eastern $n = 269$; Central $n = 37$; South-Eastern $n = 236$; Western $n = 120$). (B)-(C) The center line of the box plot indicates the median, the box covers the interquartile range, the whiskers cover the 90% central prediction interval (P5–P95), and black dots correspond to outliers outside this interval.

and dividing by λ, we arrive at

$$-\tau(B \cup \{e\}) + \tau(B) \leq -\tau(A \cup \{e\}) + \tau(A) \,. \tag{36}$$

From definition 1, this implies that $-\tau$ is a submodular set function. □

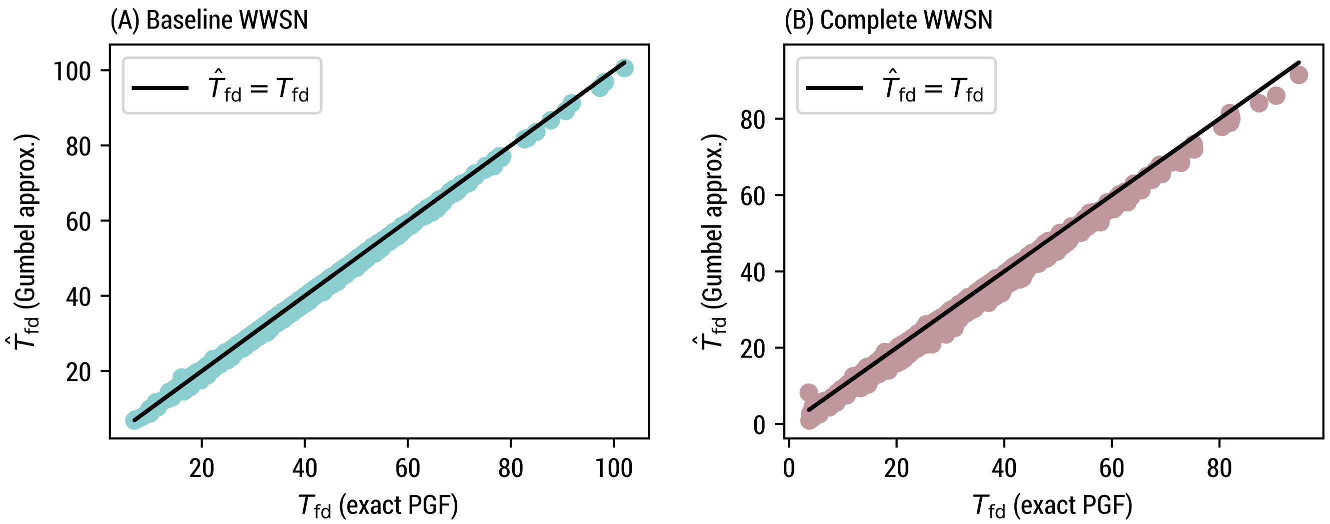

Figure S15: Comparison of the exact PGF calculation and the Gumbel approximation provided by Eq. (33) for the mean time to first detection, considering each of the 3200+ subpopulations as the origin. We use the same model parametrization as in Fig. 1. (A) We use the baseline WWSN. The Pearson correlation coefficient is > 0.999 with a two-sided P value $<10^{-16}$ ($n = 3244$), testing non-correlation. (B) We use the complete WWSN. The Pearson correlation coefficient is 0.997 with a two-sided P value $<10^{-16}$ ($n = 3244$), testing non-correlation.

3 Additional performance metrics

Our analysis primarily focused on time to first detection, but other metrics are important to assess the potential effectiveness of an outbreak response. In Fig. 3 of the main text, we provide modeling estimates for the outbreak size in the country of origin and the number of infections already dispersed internationally at the time of first detection. We use GLEAM stochastic simulations (100 per origin) for 16 different subpopulations as the source of the epidemic, all identified in Fig. S16. We reused the 4 origins of Fig. 1 (Geneva, São Paulo, Kotabaru, and Kalemie) along with 12 other subpopulations sampled at random, ensuring diversity for the time to first detection and sufficient geographical dispersion. Summary statistics for the simulation results are provided in Table S5. Note that the number of internationally dispersed infections—corresponding to international importations of latent or infectious individuals—can be zero at the time of first detection because of traveling post-infectious (but detectable) individuals.

4 Retrospective counterfactual scenarios

4.1 SARS-CoV-2 Alpha variant emergence

In the main text, we present the results of a counterfactual scenario where we would have had a global WWSN to track the international dissemination of the SARS-CoV-2 Alpha (B.1.1.7) variant. We utilize air-travel data from September 2020 to November 2020, along with the baseline WWSN of 20 sentinels (see Table S6).

This variant was first identified by health authorities in the United Kingdom and retrospective analyses trace the first identified case in Kent, South East England, on September 20, 2020 [45]. According to data published by the United Kingdom government [46], the effective reproduction number for SARS-CoV-2 between September 11 and October 30, 2020, was between 1.1 and 1.4 (90% CI) in both London and South East England. The Alpha variant was found to be more transmissible, with an estimated increased reproduction number ranging from 40 to 100% [45,

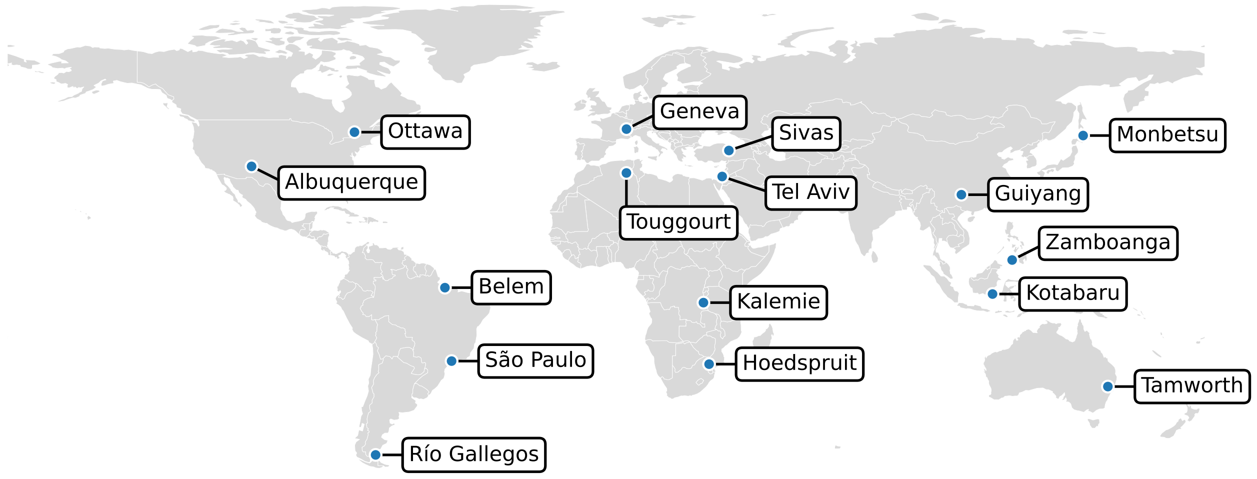

Figure S16: Epidemic origins chosen to estimate additional performance metrics with GLEAM simulations in Fig. 3.

47, 48]. In this counterfactual study, we considered a value of $\mathcal{R}_{\text{eff}}^{\text{alpha}} = 1.7$ for the Alpha variant, on the lower side of available estimates, equivalent to having an effective reproduction number of $\mathcal{R}_{\text{eff}}^{\text{ws}} = 1.1$ for the wild strain and an increased transmissibility of 55%. Since approximately 5% of positive cases were sequenced at the source [45], we considered an initial cluster of 20 infectious and 20 latent individuals on September 15. The generation time is kept fixed at 6.5 days, with a latency period of 4.5 days.

Distributions for the time to first detection in Fig. 6A are calculated using the PGF methodology and are not influenced by the wastewater sampling scheme. The geolocalization of the source (Fig. 6B) and the parameter inference (Fig. 6C), however, require us to transform distributions for the number detections to account for wastewater pool sampling at sentinel sites.

Distribution for the cumulative number of detections by a WWSN

Our PGF methodology allows us to estimate $P(d_t = d)$, the distribution for the cumulative number of detections at time t by the WWSN. However, as defined, all detectable agents traveling through a screened route could contribute to d_t *independently*. While this can be a reasonable assumption for individual testing, this is not the case for wastewater sampling, where it is not usually feasible to identify precisely the number of detectable individuals in an aircraft—we only get a binary *yes* or *no* answer to whether or not the pathogen was detected. Depending on how the testing is performed—on individual aircraft, or pooled sampling of multiple aircraft at a triturator—we would obtain different results for the cumulative number of detections.

We assume the pooled testing of all aircraft within a day at each sentinel airport. To take into account the potential copresence on the same day of detectable individuals traveling to the same destination, we need to define the probability $P(\tilde{d}|d)$, where $\tilde{d}$ represents the cumulative "wastewater" detections and d the cumulative "individual" detections we would obtain if we were able to detect each individual independently. Then we get the adjusted distribution

$$P(\tilde{d}) = \sum_d P(\tilde{d}|d)P(d) \; . \tag{37}$$

We evaluate $P(\tilde{d}|d)$ by first characterizing $q_{\nu,t}$, the relative probability an individual detection happens at sentinel ν and at time t. In general, $q_{\nu,t} \propto \langle d^*_{\nu,t} \rangle$, where $\langle d^*_{\nu,t} \rangle$ is the mean number of individual detections at sentinel ν and *incident* on time t—not the cumulative detections. The ratio $\langle d^*_{\nu,t} \rangle / \langle d^*_{\nu',t} \rangle$ is very stable in the early phase of

Table S5: Summary statistics for the outbreak size in the country of origin and the number of infections disseminated internationally at the time of first detection. For each epidemic origin, we report results for $n = 100$ simulations. P5 and P95 correspond to the 5th and 95th percentiles respectively.

	Source country outbreak size				International dissemination			
Origin	P5	median	mean	P95	P5	median	mean	P95
Geneva	78.4	518.0	734.2	2316.0	4.0	31.5	44.5	121.1
Tel Aviv	107.9	1417.5	2019.7	5915.4	2.0	20.5	29.6	77.0
Ottawa	1058.4	12049.0	15147.3	41843.6	2.0	30.0	42.2	129.1
São Paulo	993.2	12969.0	20946.4	52916.4	2.0	15.0	21.4	60.1
Albuquerque	2728.8	14847.0	20898.8	55591.9	1.0	17.0	27.4	81.4
Tamworth	1215.6	15766.5	19328.1	43192.3	2.0	13.5	19.2	56.0
Hoedspruit	7775.8	68722.5	87259.1	199986.6	1.0	9.5	12.1	32.0
Monbetsu	5297.2	29462.0	31424.2	66446.1	0.0	8.0	11.0	27.6
Río Gallegos	6988.4	31014.5	32105.8	65904.1	3.0	21.0	28.4	76.2
Zamboanga	7271.6	64520.0	83902.4	212207.8	1.0	9.5	12.6	35.0
Sivas	10194.1	86696.0	118694.5	311541.3	5.0	27.5	48.1	148.1
Guiyang	25848.3	167158.0	230677.2	805698.5	0.0	11.0	15.2	44.1
Belem	17910.3	177084.5	256976.2	692033.9	2.0	28.0	37.0	90.3
Touggourt	27902.6	180264.0	181945.0	401637.9	0.0	8.5	13.0	42.2
Kotabaru	58195.2	328464.0	344369.7	683204.4	1.0	8.5	12.2	37.0
Kalemie	542712.7	1198554.0	1354666.4	2533491.4	46.0	89.5	88.1	126.1

an outbreak for any pair of airports (ν,ν'). Therefore, $\langle d^*_{\nu,t}\rangle \sim A(\nu)e^{\lambda t}$, where λ is the growth rate at the source and $A(\nu)$ is the relative propensity of detection at each sentinel airports in the early phase. After normalization, we encapsulate all probabilities in a vector $\boldsymbol{q}$. Secondly, we assume that the number of *incident* detections for each sentinel and day $d^*_{\nu,t}$ ($\boldsymbol{d}^*$ in vector format) is distributed according to a multinomial $P(\boldsymbol{d}^*)$ with parameters d (the sum of all $\boldsymbol{d}^*$) and $\boldsymbol{q}$. The total number of *wasterwater* detections corresponds to the number of nonzero counts in $\boldsymbol{d}^*$, and thus $P(\tilde{d}|d)$ is obtained by summing $P(\boldsymbol{d}^*)$ over all configurations such that there are a total of $\tilde{d}$ nonzero counts. While this is technically a difficult combinatorial task, this can be carried out efficiently numerically (see Ref. [49]).

In Fig. S17(A), we show examples of time series for the cumulative number of detections at all sentinels of the WWSN generated by GLEAM. In Fig. S17(B), we illustrate the importance of accounting for wastewater pooled sampling when the number of detections is sufficiently large; the number of wastewater detections is effectively reduced due to the copresence of detectable individuals transiting through as sentinel during the same day.

Geolocalization of the source

Even without information about which flight paths led to detections, it is possible to recover information about the location of the source of an epidemic. Indeed, we can construct a likelihood $P(\tilde{\boldsymbol{d}}|\text{source} = l) \equiv P(\tilde{\boldsymbol{d}}|l)$ for the probability to have observed the wastewater detections $\tilde{\boldsymbol{d}} = (\tilde{d}_\nu)_{\nu\in\mathcal{S}}$ at the sentinel airports.

First, let us ignore wastewater pool sampling and consider individual detections $\boldsymbol{d} = (d_\nu)_{\nu\in\mathcal{S}}$ and the associated likelihood function $P(\boldsymbol{d}|l)$. Then $P(\boldsymbol{d}|l)$ can be modeled by a multinomial distribution with parameter $d = \sum_\nu d_\nu$ and probability vector $\boldsymbol{q} = (q_\nu)_{\nu\in\mathcal{S}}$, where $q_\nu \propto \langle d_\nu\rangle$, the expected number of detections at sentinel ν when we have d detections. In practice, we approximate q_ν by the expected number of detections at each sentinel when we have a first detection.

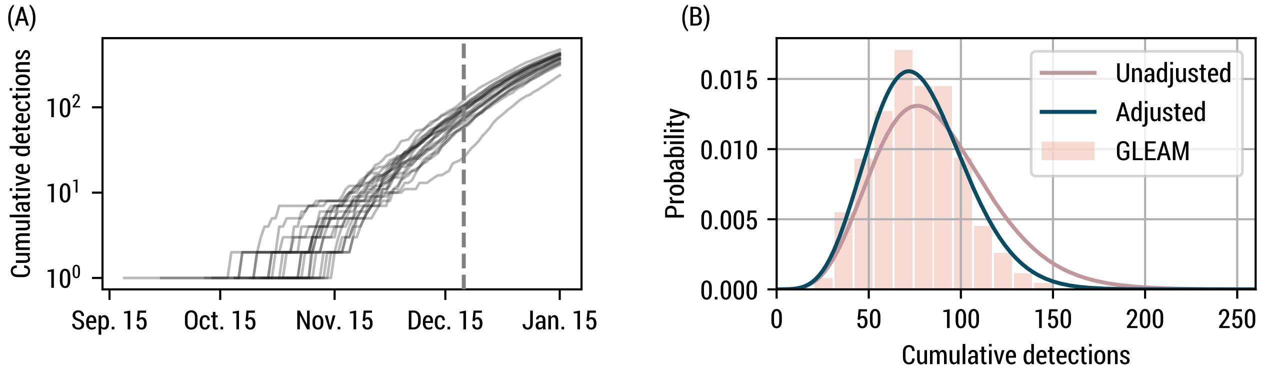

Figure S17: Cumulative number of wastewater detections for the Alpha variant counterfactual study. (A) Example of 20 detection time series from GLEAM simulations. The vertical dashed line indicates December 20, 2020. (B) Distribution for the cumulative number of detections on December 20, 2020, from 1250 GLEAM simulations and the PGF methodology, with and without adjustment for wastewater pool sampling.

To account for wastewater pool sampling, we transform the likelihood as

$$P(\tilde{\boldsymbol{d}}|l) = \sum_{\boldsymbol{d}} P(\tilde{\boldsymbol{d}}, \boldsymbol{d}|l) = \sum_{\boldsymbol{d}} P(\tilde{\boldsymbol{d}}|\boldsymbol{d}, l) P(\boldsymbol{d}|l) \; . \tag{38}$$

Assuming that $P(\tilde{\boldsymbol{d}}|\boldsymbol{d}, l)$ is concentrated around its mean, we can use the following approximation

$$P(\tilde{\boldsymbol{d}}|l) \approx \sum_{\boldsymbol{d}} \delta_{\boldsymbol{d},\boldsymbol{b}} P(\boldsymbol{d}|l) = P(\boldsymbol{b}|l) \; , \tag{39}$$

where $\boldsymbol{b}$ is the expected number of "individual detections" given the observed pooled wastewater detections $\tilde{\boldsymbol{d}}$, rounded to the nearest integer.

Using the likelihood in Eq. (39), we compute the following posterior distribution for the source

$$P(l|\tilde{\boldsymbol{d}}) \propto P(\tilde{\boldsymbol{d}}|l) P(l) \; , \tag{40}$$

where $P(l)$ is a prior distribution on the source location. In this work, we consider a uniform prior $P(l) = \text{const}$. In Fig. S18, we show this posterior distribution for at least 10 wastewater detections, averaged over 1250 simulations. We see that most of the posterior density is concentrated in Europe, and especially in the United Kingdom. The same simulations and posterior distributions are used to assess the geolocalization capacities of a WWSN in Fig. 6, as detections accumulate at the sentinels.

Characterization of the growth dynamics

The time series of cumulative detections can also be utilized to estimate key epidemic parameters. For generic parameter inference, we use the following approach. Given the observed cumulative number of wastewater detections $\tilde{d}$ and $\tilde{d}'$ at different time t and t', we calculate the posterior distribution over $\boldsymbol{\theta}$ (the parameters) using

$$P(\boldsymbol{\theta}|\tilde{d}', \tilde{d}) \propto P(\tilde{d}', \tilde{d}|\boldsymbol{\theta}) P(\boldsymbol{\theta}) \; . \tag{41}$$

To compute more efficiently the joint likelihood $P(\tilde{d}', \tilde{d}|\boldsymbol{\theta})$, we evaluate the joint cumulants from the CGF and employ the method of moments with a negative multinomial distribution.

In Fig. 6C of the main text, we jointly infer the epidemic start date and the increased transmissibility of the Alpha variant with respect to the SARS-CoV-2 wild strain. We use a flat prior $P(\boldsymbol{\theta}) = \text{const.}$, between 9% and 109% for

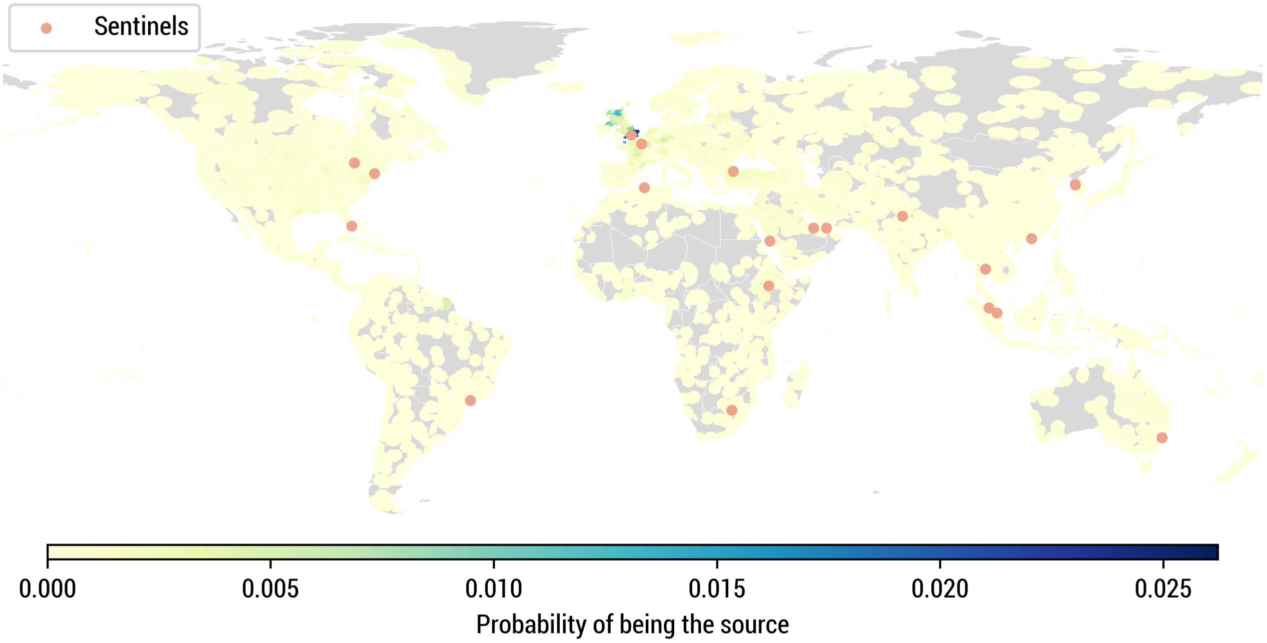

Figure S18: Geolocalization of the source for the SARS-CoV-2 Alpha variant counterfactual scenario. We show the posterior distribution over subpopulations averaged over 1250 GLEAM simulations, when ≥ 10 cumulative wastewater detections have occurred. We use the same model parametrization as in Fig. 6 for the geolocalization.

the increased transmissibility ($\mathcal{R}_{\text{eff}}^{\text{alpha}} \in [1.2, 2.3]$) and between July 12 and October 20, 2020, for the starting date (cluster of 20 latent and 20 infectious). In Fig. S19, we also illustrate a subset of the posterior distributions from individual time series.

4.2 SARS-CoV-2 (wild strain) emergence

To assess the potential effectiveness of a WWSN in early detection and response to a pandemic situation, we analyze a second hypothetical scenario: the operation of a WWSN prior to the onset of the COVID-19 pandemic. This counterfactual study utilizes air-travel data from December 2018 to February 2019, along with the baseline WWSN of 20 sentinels (see Table S6).

Calibration on importations

Considering the high uncertainty at the beginning of the pandemic, we first calibrate our model to available data. To do so, we use the same approach as in Refs. [5, 6], i.e., we calibrate our model on the number of international importations until January 23rd, 2020, when travel restrictions were imposed.

Only a fraction of importations are identified at the destination, notably because of asymptomatic individuals—we considered a 40% rate of asymptomatic individuals [50]—, but also because of different levels of capacity for detection. To account for the heterogeneity in case detection, we stratify countries into three groups based on the second component of the Global Health Security Index: high ($\geq$80th percentile), low ($\leq$20th percentile), and medium (the rest) surveillance capacity. We then use the estimates provided in Ref. [51] to assign a probability of detection relative to Singapore, which has had strong epidemiological surveillance in past infectious disease outbreaks including the COVID-19 pandemic. We used a 60% probability of detection for symptomatic individuals in Singapore, and then countries in the high, medium, and low categories were assigned relative capacities corresponding to 40%, 37%, and 11% compared to Singapore.

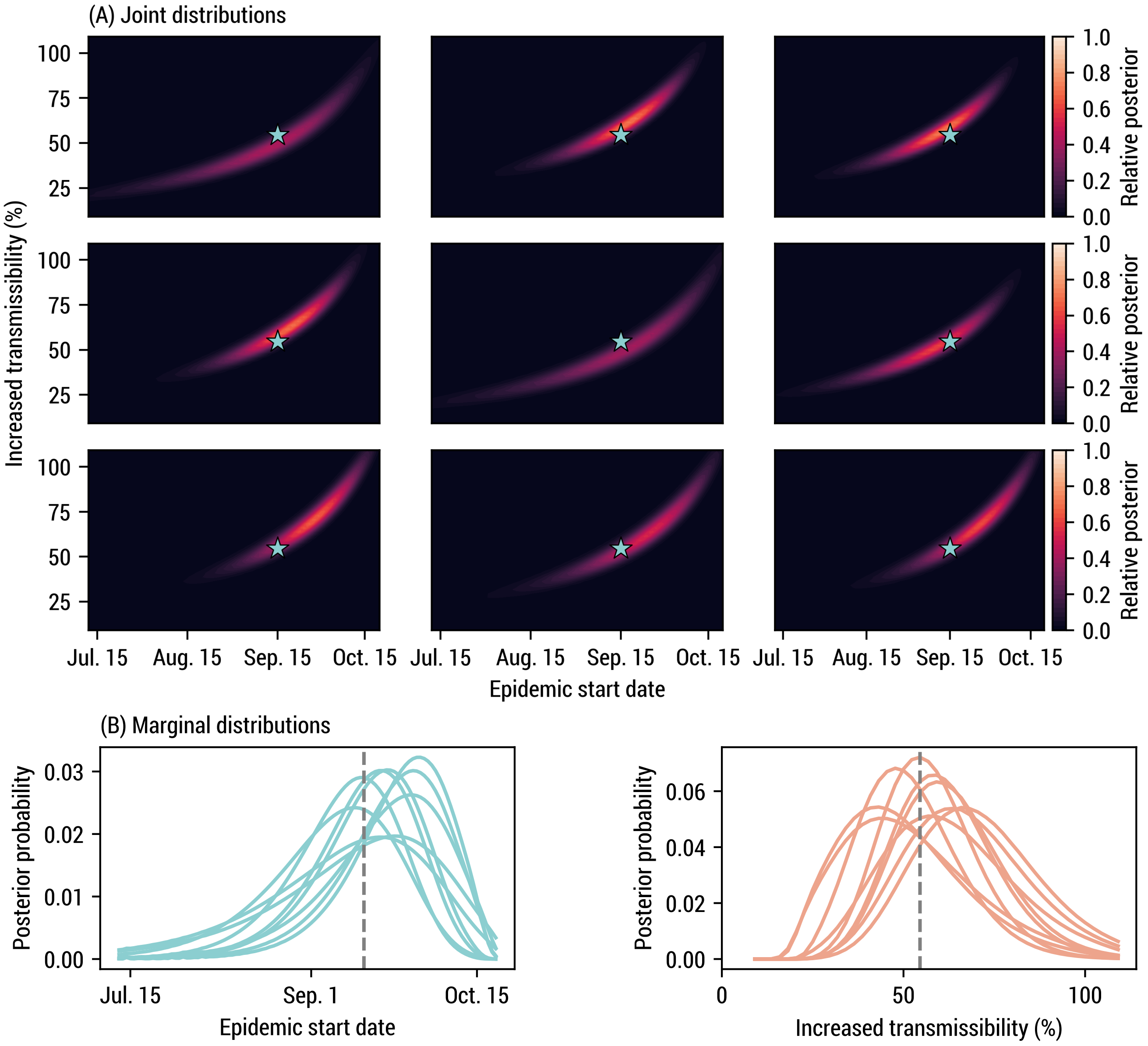

Figure S19: Examples of posterior distributions obtained for a subset of detection time series ($n = 9$) used to construct the average posterior distribution in Fig. 6C. (A) Joint posterior distributions. The blue star represents the ground truth (55% increased transmissibility and September 15, 2020, as the start date). (B) Marginal distributions, integrating over each axis. The vertical dashed line indicates the ground truth.

To calibrate our model, we compute the distribution $P(E|\boldsymbol{\theta})$ for the number of importations E given the parameters $\boldsymbol{\theta}$ of the model—in the present case, $\boldsymbol{\theta}$ corresponds to the start date and the basic reproduction number. This likelihood is used to evaluate the posterior distribution $P(\boldsymbol{\theta}|E) \propto P(E|\boldsymbol{\theta})$. We use a uniform prior between November 15th and December 1st, 2019 for the starting date with an initial cluster of 10 infectious and 10 latent individuals in Wuhan, consistent with estimates placing the index case mid-October to mid-November [52]. We also consider a uniform prior distribution on $\mathcal{R}_0$ between 2 and 2.9. The generation time is kept fixed at 6.5 days, with a latency period of 4.5 days.

In Fig. S20(A), we show the posterior distribution obtained for $\mathcal{R}_0$ and the starting date using as evidence 69

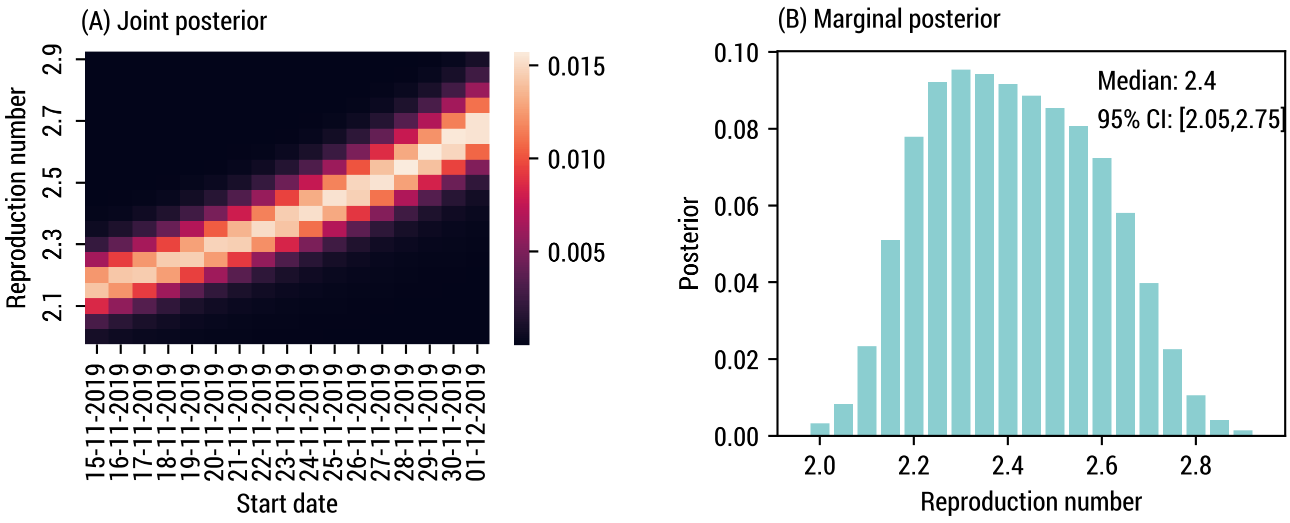

Figure S20: Calibration on international importations for the emergence of SARS-CoV-2 (wild strain). We assume an initial cluster of 10 infectious and 10 latent individuals, a generation time of 6.5 days, and a detectable period of 12.7 days. (A-B) Posterior distributions given 69 international COVID-19 cases with a travel history and an arrival date before or on Jan. 23, 2020.

identified importations by Jan. 23, 2020 (see Table S1 in the Supplementary Material of Ref. [5]). Summing the joint posterior over the plausible starting date, we obtain the marginal posterior distribution on $\mathcal{R}_0$ in Fig. S20(B), with a median of 2.4 (95% CI, 2.05–2.75). The resulting median doubling time is 4.05 days (95% CI, 3.35—5.15), broadly consistent with other estimates [5, 6, 13, 15, 16].

Results

In Fig. S21, we present similar results as in Fig. 6, but for the emergence of SARS-CoV-2 in Wuhan, demonstrating the robust early warning and situational awareness capacities of a global WWSN.

We use the joint posterior of Fig. S20(A) to compute the posterior predictive distribution for the time to first detection in Fig. S21(A). We find that even with a low detection rate (4%), there is a 54% probability that an international case outside China would have been detected earlier than the first reported case in Thailand (January 13th), with a median time to first detection on January 12th. With a 16% detection rate—more in line with estimates for a triturator sampling scheme capturing all international inbound flights—the probability to have detection prior to January 13th is 91% and the median time to first detection is on January 4th.

This hypothetical scenario presupposes the capability of detecting SARS-CoV-2 in aircraft wastewater. The key point, however, is that an operational WWSN has the potential to significantly expedite the detection of international pathogen introductions. Any reduction in the time taken to sequence the genome of emerging pathogens would directly correlate to earlier detections. Furthermore, frozen samples can be stored and analyzed in the future.

Similarly to the Alpha variant retrospective study, we use synthetic time series data from GLEAM to illustrate how we can evaluate the source and key epidemic parameters. We fix the basic reproduction number to $\mathcal{R}_0 = 2.4$ and an initial cluster of 10 infectious and 10 latent individuals on November 23, 2019, based on the joint posterior in Fig. S20(A). We obtain the posterior distribution over subpopulations as a function of the number of wastewater detections and aggregate the posterior distribution at the continent and country level in Fig. S21(C). We find reliable geolocalization capacities at the country level after about 10 detections, with a median posterior probability higher

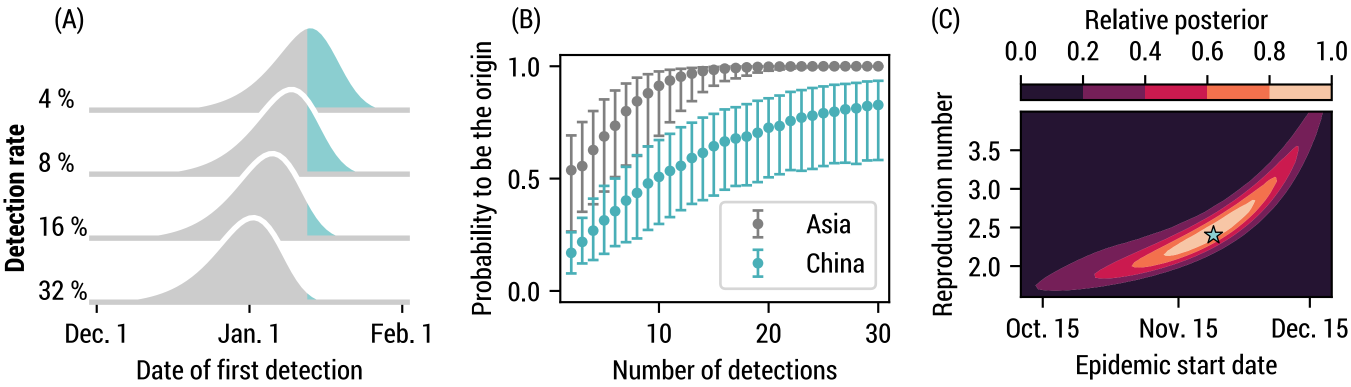

Figure S21: A global WWSN would provide an early warning system for international spreading and timely inferential capabilities. We consider a counterfactual scenario of the emergence of COVID-19 where a global WWSN would have been available. We use the baseline surveillance system consisting of 20 sentinels (see Table S6). We calibrate our model on international importations with an arrival date on or before Jan. 23 2020. (A) Distributions for the time to first detection with varying detection rates. The blue portion corresponds to dates after the first international case was reported in Thailand on January 13 [53]. (B)-(C) Inference experiment using data generated by the mechanistic GLEAM model with $\mathcal{R}_0 = 2.4$ and a start date of November 23 for the initial cluster. (B) Geolocalization of the source as more detections cumulate. We compute the posterior distribution for the origin of the epidemic based on the detection counts at each sentinel. Geolocalization of the source as more detections cumulate. We compute the posterior distribution for the origin of the epidemic based on the detection counts at each sentinel. The markers indicate the median posterior value and the error bars cover the interquartile range obtained from $n = 1200$ detection time series. (C) Joint posterior distribution on $\mathcal{R}_0$ and the start date, averaged over 59 detection time series. The blue star indicates the ground truth for the simulation experiment.

than 50%.

We also infer the basic reproduction number and the epidemic start date using the cumulative number of detections up until January 16 as the first observation $\tilde{d}$, and the cumulative number of detections between January 16 and January 23 as the second observation $\tilde{d}'$. We use a flat prior $P(\boldsymbol{\theta}) = \text{const.}$, between 1.5 and 4 for $\mathcal{R}_0$ and between October 5th and December 20th for the starting date (cluster of 10 latent and 10 infectious). The posterior distribution averaged over 59 time series is illustrated in Fig. S21(C). This average posterior indicates a median basic reproduction number of 2.4 (90% CI, 1.75–3.55), and a median starting date on November 20th (90% CI, Oct. 17–Dec. 14), illustrating the timely analytics a global WWSN could provide about the growth dynamics of emerging outbreaks.

5 Airport table for the sentinel surveillance system

We report in Table S6 the list of sentinel airports for the baseline WWSN and the different optimization schemes (volume, entropy, greedy) when considering all subpopulations as equiprobable sources of an epidemic. Each airport is identified by its IATA code. We additionally list the sentinels obtained by the greedy approach in the context of targeted optimization, assuming all subpopulations within a specific continent as equiprobable sources of an epidemic, but all subpopulations outside the continent are ignored.

References

1. Balcan, D. *et al.* Multiscale mobility networks and the spatial spreading of infectious diseases. *Proc. Natl. Acad. Sci. U.S.A.* **106,** 21484–21489 (2009).
2. Balcan, D. *et al.* Modeling the spatial spread of infectious diseases: The GLobal Epidemic and Mobility computational model. *J. Comput. Sci.* **1,** 132–145 (2010).
3. Pastore-Piontti, A. *et al.* in *Mathematical and Statistical Modeling for Emerging and Re-emerging Infectious Diseases* (eds Chowell, G. & Hyman, J. M.) 39–56 (Springer International Publishing, Cham, 2016).
4. Zhang, Q. *et al.* Spread of Zika virus in the Americas. *Proc. Natl. Acad. Sci. U.S.A.* **114,** E4334–E4343 (2017).
5. Chinazzi, M. *et al.* The effect of travel restrictions on the spread of the 2019 novel coronavirus (COVID-19) outbreak. *Science* **368,** 395–400 (2020).
6. Davis, J. T. *et al.* Cryptic transmission of SARS-CoV-2 and the first COVID-19 wave. *Nature* **600,** 127–132 (2021).
7. Gridded Population of the World (GPW). Socioeconomic Data and Applications Center (SEDAC). https://sedac.ciesin.columbia.edu/data/collection/gpw-v4, Accessed on September 12, 2023.
8. Mistry, D. *et al.* Inferring high-resolution human mixing patterns for disease modeling. *Nat. Commun.* **12,** 323 (2021).
9. Zipf, G. K. The P1 P2/D Hypothesis: On the Intercity Movement of Persons. *Am. Sociol. Rev.* **11,** 677–686 (1946).
10. Simini, F. *et al.* A universal model for mobility and migration patterns. *Nature* **484,** 96–100 (2012).
11. Wearing, H. J., Rohani, P. & Keeling, M. J. Appropriate Models for the Management of Infectious Diseases. *PLOS Med.* **2,** 621–627 (2005).
12. Krylova, O. & Earn, D. J. D. Effects of the infectious period distribution on predicted transitions in childhood disease dynamics. *J. R. Soc. Interface* **10,** 20130098 (2013).
13. Riou, J. & Althaus, C. L. Pattern of early human-to-human transmission of Wuhan 2019 novel coronavirus (2019-nCoV), December 2019 to January 2020. *Eurosurveillance* **25,** 2000058 (2020).
14. Li, Q. *et al.* Early Transmission Dynamics in Wuhan, China, of Novel Coronavirus–Infected Pneumonia. *N. Engl. J. Med.* **382,** 1199–1207 (2020).
15. Adhikari, S. P. *et al.* Epidemiology, causes, clinical manifestation and diagnosis, prevention and control of coronavirus disease (COVID-19) during the early outbreak period: a scoping review. *Infect. Dis. Poverty* **9,** 29 (2020).
16. Read, J. M. *et al.* Novel coronavirus 2019-nCoV (COVID-19): early estimation of epidemiological parameters and epidemic size estimates. *Philos. Trans. R. Soc. B* **376,** 20200265 (2021).
17. Griffin, J. *et al.* Rapid review of available evidence on the serial interval and generation time of COVID-19. *BMJ Open* **10,** e040263 (2020).
18. Foladori, P. *et al.* SARS-CoV-2 from faeces to wastewater treatment: What do we know? A review. *Sci. Total Environ.* **743,** 140444 (2020).
19. Weiss, A., Jellingsø, M. & Sommer, M. O. A. Spatial and temporal dynamics of SARS-CoV-2 in COVID-19 patients: A systematic review and meta-analysis. *EBioMedicine* **58,** 102916 (2020).
20. Li, Q. *et al.* Number of COVID-19 cases required in a population to detect SARS-CoV-2 RNA in wastewater in the province of Alberta, Canada: Sensitivity assessment. *J. Environ. Sci.* **125,** 843–850 (2023).

21. Brauer, F. *et al.* Mathematical epidemiology (Springer, 2008).

22. Allen, L. J. S. An introduction to stochastic processes with applications to biology (CRC press, 2010).

23. Miller, J. C. A primer on the use of probability generating functions in infectious disease modeling. *Infect. Dis. Model.* **3,** 192–248 (2018).

24. Roberts, D. *et al.* Quantifying the impact of individual and collective compliance with infection control measures for ethical public health policy. *Sci. Adv.* **9,** eabn7153 (2023).

25. Allard, A. *et al.* Complex Systems Modeling notes. COSMO-notes. https://cosmo-notes.github.io/index.html, Accessed on March 21, 2024.

26. Noël, P.-A. *et al.* Time evolution of epidemic disease on finite and infinite networks. *Phys. Rev. E* **79,** 026101 (2009).

27. Allen, A. J. *et al.* Predicting the diversity of early epidemic spread on networks. *Phys. Rev. Res.* **4,** 013123 (2022).

28. Boudreau, M. C. *et al.* Temporal and Probabilistic Comparisons of Epidemic Interventions. *Bull. Math. Biol.* **85,** 118 (2023).

29. Johansson, M. A. *et al.* Assessing the Risk of International Spread of Yellow Fever Virus: A Mathematical Analysis of an Urban Outbreak in Asunción, 2008. *Am. J. Trop. Med. Hyg.* **86,** 349–358 (2012).

30. Lahodny, G. E. & Allen, L. J. S. Probability of a Disease Outbreak in Stochastic Multipatch Epidemic Models. *Bull. Math. Biol.* **75,** 1157–1180 (2013).

31. Johansson, M. A. *et al.* Nowcasting the Spread of Chikungunya Virus in the Americas. *PLOS ONE* **9,** e104915 (2014).

32. Mier-y-Teran-Romero, L., Tatem, A. J. & Johansson, M. A. Mosquitoes on a plane: Disinsection will not stop the spread of vector-borne pathogens, a simulation study. *PLOS Negl. Trop. Dis.* **11,** 1–13 (2017).

33. Lai, S. *et al.* Seasonal and interannual risks of dengue introduction from South-East Asia into China, 2005-2015. *PLOS Negl. Trop. Dis.* **12,** 1–16 (2018).

34. Truelove, S. *et al.* Epidemics, Air Travel, and Elimination in a Globalized World: The Case of Measles. *medRxiv* (2020).

35. Abate, J. & Whitt, W. Numerical inversion of probability generating functions. *Oper. Res. Lett.* **12,** 245–251 (1992).

36. Hébert-Dufresne, L. *et al.* Beyond R0: heterogeneity in secondary infections and probabilistic epidemic forecasting. *J. R. Soc. Interface* **17,** 20200393 (2020).

37. Gautreau, A., Barrat, A. & Barthélemy, M. Global disease spread: Statistics and estimation of arrival times. *J. Theor. Biol.* **251,** 509–522 (2008).

38. Brockmann, D. & Helbing, D. The Hidden Geometry of Complex, Network-Driven Contagion Phenomena. *Science* **342,** 1337–1342 (2013).

39. Iannelli, F. *et al.* Effective distances for epidemics spreading on complex networks. *Phys. Rev. E* **95,** 012313 (2017).

40. Chen, L. M., Holzer, M. & Shapiro, A. Estimating epidemic arrival times using linear spreading theory. *Chaos* **28,** 013105 (2018).

41. Jamieson-Lane, A. & Blasius, B. Calculation of epidemic arrival time distributions using branching processes. *Phys. Rev. E* **102,** 042301 (2020).

42. Lloyd-Smith, J. O. *et al.* Superspreading and the effect of individual variation on disease emergence. *Nature* **438,** 355–359 (2005).

43. Airline and Airport Code Search. IATA. https://www.iata.org/en/publications/directories/code-search/, Accessed on July 30, 2024.

44. Nemhauser, G. L., Wolsey, L. A. & Fisher, M. L. An analysis of approximations for maximizing submodular set functions—I. *Math. Program.* **14,** 265–294 (1978).

45. World Health Organization. COVID-19 - United Kingdom of Great Britain and Northern Ireland. https://www.who.int/emergencies/disease-outbreak-news/item/2020-DON304, Accessed on March 19, 2024.

46. Agency, U. H. S. The R value and growth rate. www.gov.uk/guidance/the-r-value-and-growth-rate, Accessed on September 15, 2023.

47. Davies, N. G. *et al.* Estimated transmissibility and impact of SARS-CoV-2 lineage B.1.1.7 in England. *Science* **372,** eabg3055 (2021).

48. Volz, E. *et al.* Assessing transmissibility of SARS-CoV-2 lineage B.1.1.7 in England. *Nature* **593,** 266–269 (2021).

49. Distribution of number of non-zero counts of a multinomial distributed set. Mathematics Stack Exchange. https://math.stackexchange.com/q/4767477, Accessed on September 12, 2023.

50. Oran, D. P. & Topol, E. J. Prevalence of Asymptomatic SARS-CoV-2 Infection. *Annals of Internal Medicine* **173,** 362–367 (2020).

51. Niehus, R. *et al.* Using observational data to quantify bias of traveller-derived COVID-19 prevalence estimates in Wuhan, China. *Lancet Infect. Dis.* **20,** 803–808 (2020).

52. Pekar, J. *et al.* Timing the SARS-CoV-2 index case in Hubei province. *Science* **372,** 412–417 (2021).

53. World Health Organization. Novel Coronavirus – Thailand (ex-China). https://www.who.int/emergencies/disease-outbreak-news/item/2020-DON234, Accessed on September 15, 2023.

Table S6: Ordered list of sentinel airports (most important first) identified by the different optimization schemes. Each airport is identified by its IATA code [43]. We use the aggregated global air-travel network from September 2022 to August 2023. We also list the (unordered) baseline sentinels as determined in the main text.

Global				Targeted (greedy)					
baseline	volume	entropy	greedy	Africa	Asia	Europe	N. America	Oceania	S. America
ADD	DXB	FRA	LHR	DXB	DXB	IST	LHR	AKL	MIA
JNB	LHR	CDG	DXB	CDG	SIN	AMS	CUN	SIN	PTY
ALG	CDG	AMS	CDG	ADD	ICN	STN	YYZ	BNE	SCL
DXB	IST	LHR	SIN	IST	BKK	AYT	CDG	LAX	MAD
DOH	AMS	IST	IST	JNB	IST	FRA	MIA	HNL	LIS
JED	SIN	MUC	FRA	LIS	HKG	LGW	YVR	SYD	LIM
LHR	FRA	BRU	MIA	JED	JED	CPH	FRA	DPS	GRU
CDG	ICN	DXB	ICN	NBO	KUL	DXB	LAX	NAN	BOG
IST	MAD	VIE	AMS	LHR	TPE	DUB	MEX	MNL	AEP
JFK	DOH	DOH	CUN	MRS	DOH	VIE	AMS	SFO	EZE
YYZ	BKK	FCO	MAD	DOH	LHR	LHR	CPH	NRT	FLL
MIA	LGW	MXP	BKK	RUN	CAI	TLV	ICN	DXB	JFK
ICN	BCN	MAN	LAX	CAI	FRA	CDG	IAH	CDG	MEX
BKK	DUB	YYZ	DOH	BRU	DMK	EVN	PVR	KUL	CDG
DEL	HKG	STN	HKG	FRA	SHJ	MUC	PUJ	HKG	CUN
SIN	JFK	ZRH	YYZ	AMS	NRT	BCN	SJD	MEL	LHR
HKG	KUL	JFK	JED	EBB	SVO	TAS	FLL	ICN	MCO
KUL	FCO	LGW	LIS	CMN	KWI	ARN	YUL	LHR	SDQ
SYD	LIS	IAD	BNE	MXP	MNL	LTN	DFW	NOU	MVD
GRU	TPE	JNB	CPH	TUN	SGN	MAN	NRT	DOH	AMS
–	VIE	MAD	PTY	LYS	AUH	FCO	JFK	GUM	FRA
–	MUC	PRG	AYT	ABJ	MED	OSS	MNL	POM	HAV
–	ORY	DUS	TPE	MAD	CGK	PMI	YYC	BKK	PUJ
–	JED	ADD	KUL	BOM	CDG	DUS	PTY	CHC	FCO
–	YYZ	WAW	ADD	DZA	MCT	MAD	EWR	CNS	IAH
–	CPH	ORD	LGW	LPA	DEL	GYD	MBJ	YVR	YYZ
–	STN	YUL	NRT	KGL	KIX	ZRH	GDL	DEL	ATL
–	ZRH	EDI	JFK	ACC	DUS	BER	ATL	HND	BCN
–	TLV	DUB	FCO	RUH	SYD	FRU	BOG	SGN	ASU
–	CAI	BCN	AKL	BCN	SAW	AGP	MCO	ZQN	SJO
–	MAN	CPH	MNL	FCO	RUH	LIS	MAD	VLI	VVI
–	MIA	ZAG	JNB	DAR	MFM	BRU	HND	KIX	MXP
–	MXP	BER	MUC	LFW	AMS	SVO	DUB	TPE	UIO
–	BRU	CPT	GRU	LGW	HAN	ALC	MUC	WLG	LAX
–	CUN	LAX	DUB	DSS	LED	DYU	TPE	SEA	ZRH
–	NRT	BUD	CAI	KWI	PUS	MXP	ORD	CGK	EWR
–	MNL	MIA	MEX	DEL	BOM	CRL	FCO	SUV	IST
–	PMI	CRL	YVR	CPT	MEL	OSL	GRU	PVG	DXB
–	AUH	TLV	STN	KRT	AMM	OPO	LAS	OOL	GYE
–	LAX	BHX	SYD	LOS	DAC	LBD	DOH	FRA	DOH
